# PLVAP and GKN3 Are Two Critical Host Cell Receptors Which Facilitate Japanese Encephalitis Virus Entry Into Neurons

**DOI:** 10.1038/s41598-018-30054-z

**Published:** 2018-08-06

**Authors:** Sriparna Mukherjee, Nabonita Sengupta, Ankur Chaudhuri, Irshad Akbar, Noopur Singh, Sibani Chakraborty, Amol Ratnakar Suryawanshi, Arindam Bhattacharyya, Anirban Basu

**Affiliations:** 1 National Brain Research Centre, Manesar, Haryana 122052 India; 2 Immunology Lab, Department of Zoology, University of Calcutta, 35, Ballygunge Circular Road, Kolkata, 700019 India; 3 West Bengal State University, North 24 Parganas, Barasat, Kolkata, 700126 India; 4 Clinical Proteomics, Institute of Life Sciences, Bhubaneswar, Odisha 751023 India; 5 Present Address: Microbiology and Cell Biology, Indian Institute of Science, CV Raman Avenue, Bangalore, Karnataka 560012 India

## Abstract

Japanese Encephalitis Virus (JEV), a globally important pathogen, belongs to the family *Flaviviridae*, is transmitted between vertebrate hosts by mosquitoes, principally by *Culex tritaeniorhynchus*. The E-glycoprotein of the virus mediates its attachment to the host cell receptors. In this study, we cloned and purified JEV E-glycoprotein in pET28a vector using *E. coli* BL21 (DE3) cells. A pull down assay was performed using plasma membrane fraction of BALB/c mouse brain and E-glycoprotein as a bait protein. 2-Dimensional Gel Electrophoresis based separation of the interacting proteins was analyzed by mass spectrometry. Among all the identified partners of E-glycoprotein, PLVAP (Plasmalemma vesicle associated protein) and GKN3 (Gastrokine3) showed significant up-regulation in both JEV infected mouse brain and neuro2a cells. In-silico studies also predicted significant interaction of these receptors with E-glycoprotein. Additionally, overexperssion and silencing of these receptors resulted in increase and reduction in viral load respectively, suggesting them as two critical cellular receptors governing JEV entry and propagation in neurons. In support, we observed significant expression of PLVAP but not GKN3 in post-mortem autopsied human brain tissue. Our results establish two novel receptor proteins in neurons in case of JEV infection, thus providing potential targets for antiviral research.

## Introduction

Japanese Encephalitis Virus belonging to the family *Flaviviridae*, is a major cause of epidemic encephalitis worldwide especially in eastern and south-eastern Asia covering a population of approximately 3 billion^1^. Nearly 1% of the virus infected human population develops encephalitis symptoms; out of which 20–30% cases are fatal while 30–50% survivors develop consequential neurological damage^2^. Like all other viruses, JEV infection in a host requires interaction of viral attachment proteins and cellular membrane proteins. The 52 kDa JEV–E glycoprotein mediates viral attachment to the host cell membrane proteins followed by membrane fusion. E glycoprotein has three antigenic domains^3^. Domain I (DI) contains 9 beta-barrels present in between domain II (DII) and globular domain III (DIII). Domain III is found to be present at the C-terminus of the protein and is connected to DI by a short peptide linker. DIII mediates the main interaction of the E-glycoprotein with the host cell membrane. Immunoglobulin like domain DIII is reported to play a major role in membrane protein interaction in cases of Dengue and Tick borne encephalitis viruses^4,5^.

The other flaviviruses like West Nile Virus and Dengue have been shown to enter host cells via clathrin mediated endocytosis^6,7^. Several studies show clathrin mediated endocytic internalization in case of JE virus too^8^. Lipid rafts are reported to be important in JEV entry into neural stem/progenitor cells through activation of Phosphoinositide 3′ Kinase/Akt signaling^9^. However, clathrin independent mechanisms are also responsible for JEV entry into fibroblasts and neuroblastoma cells^10^. Heat shock protein 70 (HSP70) has been shown to be a E-glycoprotein interactor in neuro2a cells^11^. Endoplasmic reticulum chaperone GRP78 is also an important molecule responsible for JEV internalization into neurons^12^. The other proteins taking part in JEV entry into host cells are laminin receptors and α5β3 integrin^13–15^. Although, JEV infects a broad spectrum of host cells, the major membrane receptors on the brain epithelium critical for viral entry still remain largely unknown. Hence we aimed to apply a pull down based proteomic approach to identify mouse brain membrane receptor proteins of JEV E-glycoprotein.

In the present study, JEV E-glycoprotein gene was cloned and expressed in *E. coli* BL21 (DE3) strain followed by purification through Ni-NTA beads. Simultaneously, plasma membrane fraction of 3–4 week old BALB/c mouse brain was extracted and a pull down analysis was performed using JEV E-glycoprotein as a bait protein which was then followed by 2-DE (2-dimensional gel electrophoresis) separation and mass spectrometry. Amongst the identified proteins, PLVAP (Plasmalemma vesicle-associated protein) and GKN3 (Gastrokine 3) receptor proteins were found to be significantly present in the membrane fraction of mice brain following JEV infection. We also found their presence in mouse neuro2a cell membrane, primary cortical neurons and SH-SY5Y cells at earlier time points of viral infection. Furthermore, silencing these proteins in mouse neuro2a cells prevented the viral RNA production as well as translation of viral proteins. Upon their overexperssion, viral RNA replication and protein translation were increased. In a parallel study, we found higher expression of PLVAP in basal ganglia region of autopsied human brain tissue of JE cases when compared to age matched controls of accidental injury cases. Together, our findings suggest PLVAP and GKN3 receptor proteins to be critical host factors governing JEV internalization into neurons.

## Results

### JEV E-glycoprotein interacting partners in the mouse brain epithelium

**Figure 1:**
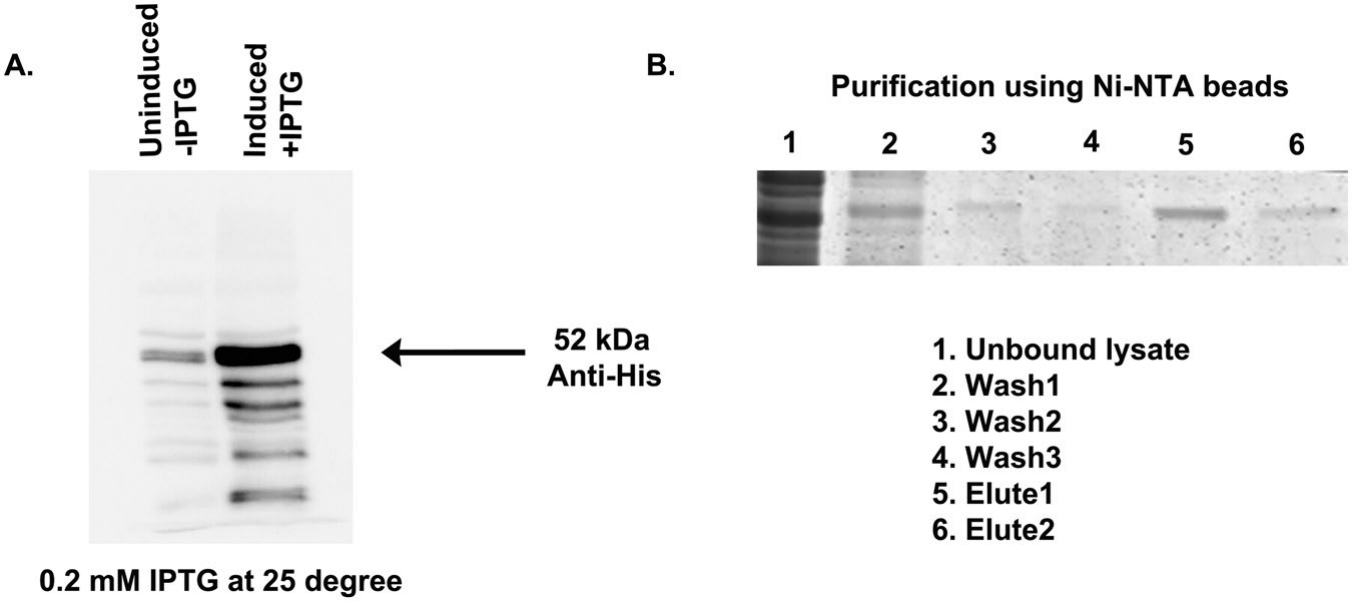
Induction and purification of JEV E-glycoprotein from *E. coli* BL21 (DE3) strain. (**A**) *E. coli* BL21 (DE3) containing E-glycoprotein fragment was induced with 0.2 mM IPTG at 25 °C. Significant amount of His-tagged E glycoprotein was found at 6 hrs. post induction. (**B**) 200 µg of bacterial protein was mixed with Ni-NTA resin at RT for 45 min. Unbound lysate and subsequent washes were checked for protein loss. The clear single band in the elute fraction indicates purification of E-glycoprotein from bacterial pellet. Data is representative of three independent experiments.

**Figure 2:**
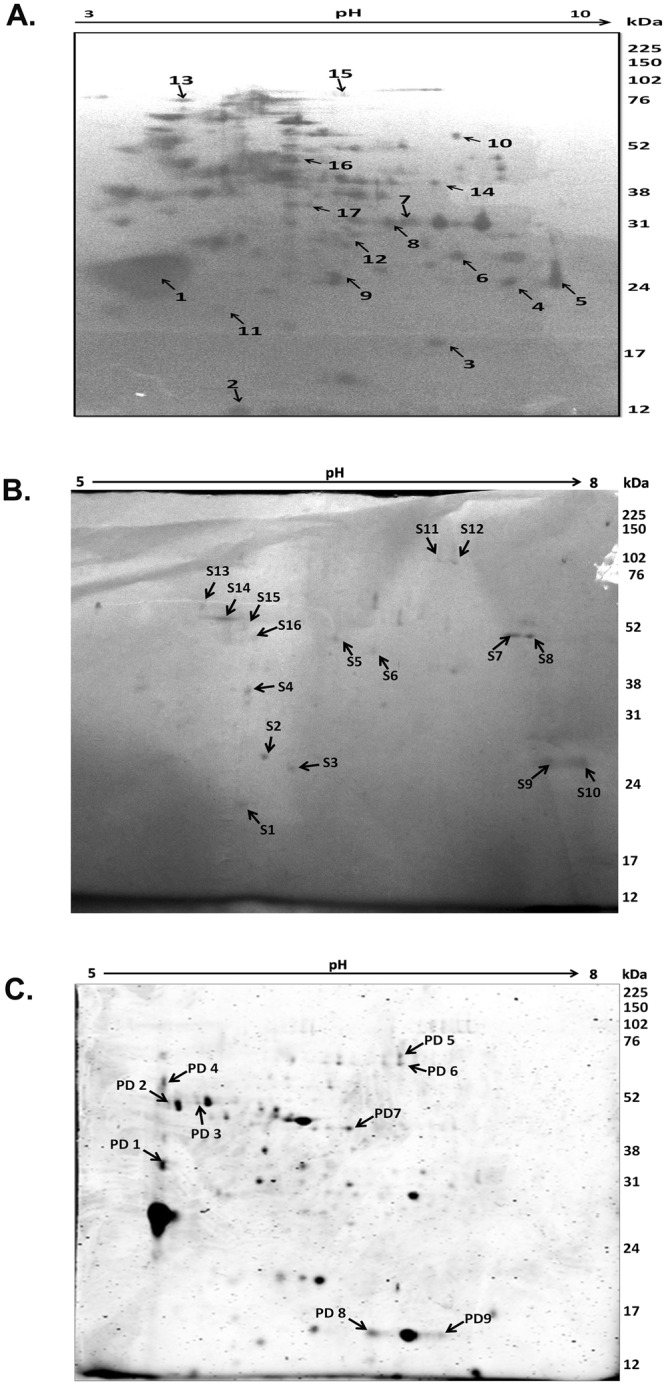
Proteomic pull down analysis of the brain membrane proteins using JEV E-glycoprotein as bait protein. (**A**) Silver staining of interacting proteins on a 12% polyacrylamide gel on an IPG strip of pH 3–10. (**B**) Silver staining of interacting proteins on a 12% polyacrylamide gel on an IPG strip of pH 5–8. (**C**) Coomassie Blue staining of interacting proteins on a 12% polyacrylamide gel on an IPG strip of pH 5–8. Spots on biological replicate experiments were marked, excised and analyzed by MALDI/TOF followed by database searches. Spots are labeled on the gel according to the numbers mentioned in Table 1.

E-glycoprotein induction was standardized at different concentrations of IPTG and at different temperatures (data not shown).   Protein expression was finally induced at 25 °C with 0.2 mM IPTG for 6 hrs. (Fig. 1A, Fig. S1). Affinity pull down analysis was performed using JEV E-glycoprotein of GP78 strain (mouse adapted) as a bait protein to identify the interacting proteins in the mouse brain membrane. Briefly 1 mg of membrane protein was incubated with 5 mg of purified His-tagged E-glycoprotein. The purity of the membrane fraction was tested by immunoblot using Caveolin and lactate dehydrogenase before proceeding with the pull down experiment (Fig. S2). After separation of the proteins by 2-DE, both silver staining (Fig. 2A,B) and coomassie staining (Fig. 2C) were done to cover a broad range of host proteins interacting with JEV E-glycoprotein. Spots that were common in biological replicate sets were identified and excised for identification by mass spectrometry. Identified proteins are enlisted in Table 1.

**Table 1:** Identification of membrane proteins.

Spot No.	Protein accession no.	Protein mass	Protein score	Protein description
**Proteins identified from silver stained gels pH 3–10**				
1	DYHC1_MOUSE	534447	45	Cytoplasmic dynein 1 heavy chain 1 OS = Musmusculus GN = Dync1h1 PE = 1 SV = 2
2	PLVAP_MOUSE	50472	44	Plasmalemma vesicle-associated protein OS = MusmusculusGN = Plvap PE = 2 SV = 1
3	ARFP2_MOUSE	37863	50	Arfaptin-2 OS = Musmusculus GN = Arfip2 PE = 2 SV = 2
4	PCM1_MOUSE	230131	45	Pericentriolar material 1 protein OS = Musmusculus GN = Pcm1 PE = 1 SV = 2
5	AP3B1_MOUSE	123291	43	AP-3 complex subunit beta-1 OS = Musmusculus GN = Ap3b1 PE = 1 SV = 2
6	KI20B_MOUSE	204986	30	Kinesin-like protein KIF20B OS = Musmusculus GN = Kif20b PE = 1 SV = 3
7	SRC8_MOUSE	61384	35	Src substrate cortactin OS = Musmusculus GN = Cttn PE = 1 SV = 2
8	IF172_MOUSE	199191	35	Intraflagellar transport protein 172 homolog OS = Musmusculus GN = Ift172 PE = 1 SV = 1
9	MELT_MOUSE	95184	44	Ventricular zone-expressed PH domain-containing protein 1 OS = Musmusculus GN = Veph1 PE = 2 SV = 2
10	SYNE1_MOUSE	1016650	48	Nesprin-1 OS = Mus musculus GN = Syne1 PE = 1 SV = 2
11	VP13C_MOUSE	422162	40	Vacuolar protein sorting-associated protein 13C OS = Musmusculus GN = Vps13c PE = 1 SV = 2
12	BIG2_MOUSE	204562	37	Brefeldin A-inhibited guanine nucleotide-exchange protein 2 OS = Musmusculus GN = Arfgef2 PE = 1 SV = 1
13	LRAT_MOUSE	26089	38	Lecithin retinol acyltransferase OS = Musmusculus GN = Lrat PE = 1 SV = 1
14	GKN3_MOUSE	20987	37	Gastrokine-3 OS = Musmusculus GN = Gkn3 PE = 1 SV = 1
15	EXOC8_MOUSE	81668	50	Exocyst complex component 8 OS = Musmusculus GN = Exoc8 PE = 1 SV = 1
16	RBNS5_MOUSE	89292	38	Rabenosyn-5 OS = Musmusculus GN = Zfyve20 PE = 2 SV = 1
17	ANXA6_MOUSE	76294	47	Annexin A6 OS = Musmusculus GN = Anxa6 PE = 1 SV = 3
**Proteins identified from silver stained gels pH 5–8**				
S1	MYH3_MOUSE	224736	50	Myosin-3 OS = Musmusculus GN = Myh3 PE = 2 SV = 2
S2	JPH3_MOUSE	81579	42	Junctophilin-3 OS = Musmusculus GN = Jph3 PE = 1 SV = 1
S3	PRDX3_MOUSE	28337	76	Thioredoxin-dependent peroxide reductase, mitochondrial OS = Musmusculus GN = Prdx3 PE = 1 SV = 1
S4	GBB1_MOUSE	38151	131	Guanine nucleotide-binding protein G(I)/G(S)/G(T) subunit beta-1 OS = Musmusculus GN = Gnb1 PE = 1 SV = 3
S5	SPTB1_MOUSE	245897	54	Spectrin beta chain, erythrocytic OS = Musmusculus GN = Sptb PE = 1 SV = 4
S6	GLNA_MOUSE	42834	188	Glutamine synthetase OS = Musmusculus GN = Glul PE = 1 SV = 6
S7	ATPA_MOUSE	59830	674	ATP synthase subunit alpha, mitochondrial OS = Musmusculus GN = Atp5a1 PE = 1 SV = 1
S8	ATPA_MOUSE	59830	868	ATP synthase subunit alpha, mitochondrial OS = Musmusculus GN = Atp5a1 PE = 1 SV = 1
S9	KI20B_MOUSE	204986	66	Kinesin-like protein KIF20B OS = Musmusculus GN = Kif20b PE = 1 SV = 3
S10	RAD50_MOUSE	154533	49	DNA repair protein RAD50 OS = Musmusculus GN = Rad50 PE = 1 SV = 1
S11	ACON_MOUSE	86151	177	Aconitatehydratase, mitochondrial OS = Musmusculus GN = Aco2 PE = 1 SV = 1
S12	ACON_MOUSE	86151	123	Aconitatehydratase, mitochondrial OS = Musmusculus GN = Aco2 PE = 1 SV = 1
S13	ATPB_MOUSE	56265	793	ATP synthase subunit beta, mitochondrial OS = Musmusculus GN = Atp5b PE = 1 SV = 2
S14	ATPB_MOUSE	56265	670	ATP synthase subunit beta, mitochondrial OS = Musmusculus GN = Atp5b PE = 1 SV = 2
S15	QCR1_MOUSE	53446	621	Cytochrome b-c1 complex subunit 1, mitochondrial OS = Musmusculus GN = Uqcrc1 PE = 1 SV = 2
S16	NRAP_MOUSE	196716	44	Nebulin-related-anchoring protein OS = Musmusculus GN = Nrap PE = 1 SV = 3
**Proteins identified from coomassie stained gels pH 5–8**				
PD1	GFAP_MOUSE	49927	57	Glial fibrillary acidic protein OS = Musmusculus GN = Gfap PE = 1 SV = 4
PD2	GFAP_MOUSE	49927	63	Glial fibrillary acidic protein OS = Musmusculus GN = GfapPE = 1 SV = 4
PD3	GFAP_MOUSE	49927	53	Glial fibrillary acidic protein OS = Musmusculus GN = Gfap PE = 1 SV = 4
PD4	MBP_MOUSE	27151	48	Myelin basic protein OS = Musmusculus GN = Mbp PE = 1 SV = 2
PD5	SOX5_MOUSE	84265	32	Transcription factor SOX-5 OS = Musmusculus GN = Sox5 PE = 1 SV = 2
PD6	CAC1A_MOUSE	269244	36	Voltage-dependent P/Q-type calcium channel subunit alpha-1A OS = Musmusculus GN = Cacna1a PE = 1 SV = 2
PD7	DYHC1_MOUSE	534447	36	Cytoplasmic dynein 1 heavy chain 1 OS = Musmusculus GN = Dync1h1 PE = 1 SV = 2
PD8	F261_MOUSE	55385	37	6-phosphofructo-2-kinase/fructose-2,6-bisphosphatase 1 OS = Musmusculus GN = Pfkfb1 PE = 2 SV = 2
PD9	SARG_MOUSE	65451	40	Specifically androgen-regulated gene protein OS = Musmusculus GN = Sarg PE = 2 SV = 2

MS and MSMS spectra were acquired on 5800 MALDI TOF/TOF and analyzed with Protein pilot V4.0 software using MASCOT search engine from Sciex. The peak list was searched against the taxonomy *Mus musculus* at protein sequence Database: UniProtKB-SwissProt sprot_2014-04-16 (544996 sequences; 193815432 residues).

Search parameters were as follows: Trypsin digestion with one missed cleavage.

Fixed modification: carbamidomethyl (c) variable modification: oxidation (m) and the peptide mass tolerance of 100 ppm for precursor ion and mass tolerance of 0.8 Da for fragment ion with +1 charge state and instrument MALDI-TOF-TOF.

### Expression, modeling and protein-protein docking of the identified membrane proteins in mouse brain post JE virus infection

**Figure 3:**
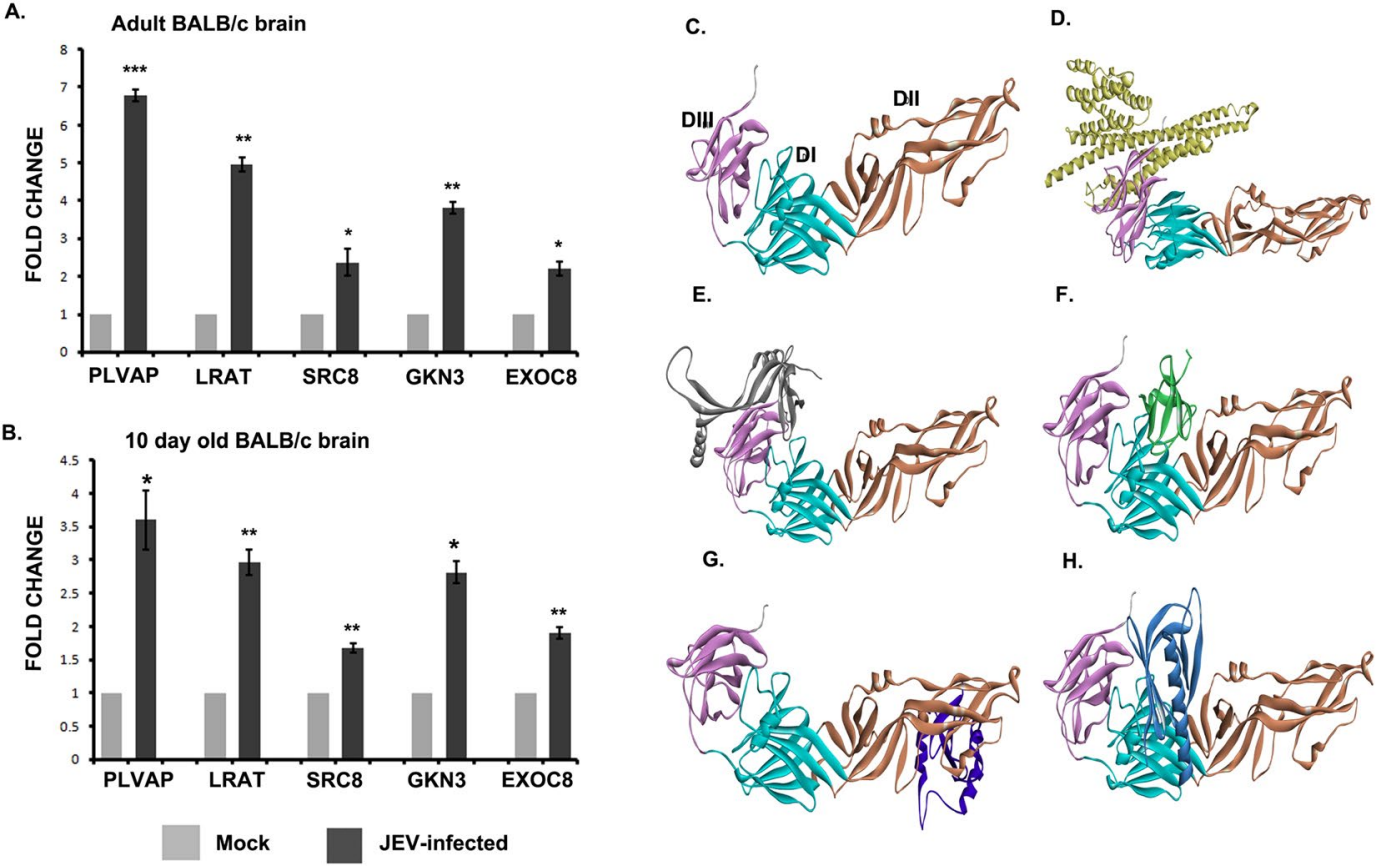
Validation and protein-protein docking of the identified membrane proteins in BALB/c mouse brain post JEV infection using qRT-PCR and ZDOCK/RDOCK programmes(**A**,**B**) JEV infection led to significant up-regulation at mRNA level of selective membrane proteins (PLVAP, LRAT, SRC8, GKN3 and EXOC8) in both adult and 10 day old BALB/c mice. (*p < 0.5, **p < 0.01, *** p < 0.001). Data is representative of three independent experiments (mean ± SD) by Student’s *t*-test. (**C**) Domain composition of JEV- E protein. DI, DII and DIII domains are marked as cyan, orange and magenta colour respectively. (**D**) Interaction of PLVAP and JEV- E. PLVAP (light yellow colour) fits into DIII domain of JEV- E. (**E**) Interaction of LRAT and JEV- E. LRAT (gray colour) fits into DIII domain of JEV- E. (**F**) Interaction of SRC8 and JEV- E. SRC8 (green colour) fits into DI domain of JEV- E. (**G**) Interaction of GKN3 and JEV- E. GKN3 (violet colour) fits into DII domain of JEV- E. (**H**) Interaction of EXOC8 with JEV- E. EXOC8 (light blue colour) fits into DI domain of JEV.

After proteomic identification of the E-glycoprotein interactome, we intended to identify the ones having differential expression in viral infection. We experimented on both infant (10 day old) and adult (3–4 weeks old) age groups of mock infected and JEV infected BALB/c mice. Animals were either mock infected with PBS or infected with virus. Animals showed encephalitic symptoms after day 5 post infection. On day 7, brain samples were collected. Expressions of identified receptor proteins were validated through qRT-PCR. Five membrane proteins namely PLVAP, LRAT, SRC8, GKN3 and EXOC8 showed notable up-regulation in both the age groups of infected animals when compared to mock (Fig. 3A,B). The three dimensional structures of these membrane proteins PLVAP, GKN3, LRAT, EXOC8, SRC8 were successfully predicted using homology modelling and ab initio method. The stereo-chemical quality of each receptor was validated by Ramachandran plot which shows above 96% residues present in the allowed region. The molecular docking of JEV-E with its corresponding five interactors PLVAP, GKN3, LRAT, EXOC8, and SRC8 was performed by using ZDOCK and RDOCK programs. The structure with lowest E_RDock score was selected to identify important amino acids at the interface region (Table S4). The interactions are depicted in Fig. 3C–H. Residues of PLVAP protein involved in interaction with JEV- E are present in domain III region with lowest E_RDock score −29.27 kcal/mol. The binding energy was calculated using Poisson-Boltzmann with non-polar surface area (PBSA) method^16^. The PLVAP -JEV- E complex formed seven hydrogen bonds and one salt bridge with binding energy of −162.99 kcal/mol (Table S5). The binding modes of LRAT on JEV- E protein were engaged with seven hydrogen bonds and three pi cation interactions. The best pose of LRAT is present at domain III of JEV envelope protein with binding energy of −156.29 kcal/mol (Table S6). The protein-protein interactions between JEV-E and SRC8 revealed six hydrogen bonds and five salt bridges with lowest E_RDock score of −30.47 kcal/mol. It was observed that the lowest energy pose of SRC8 is present at domain I region of envelope protein of JEV (Table S7) with a value of −87.14 kcal/mol. Similarly, for GKN3, the best pose with −27.85 kcal/mol E_RDock value was selected for analyzing the binding interaction network at the interface. It was packed at domain II of JEV by forming eleven hydrogen bonds, three salt bridges and one pi cation interaction with binding energy of −88.43 kcal/mol (Table S8). It was observed that binding of EXOC8 with JEV- E occurs at domain I forming seven hydrogen bonds and two salt bridges (Table S9) with a binding energy of −130.06 kcal/mol. Study of the interaction network present at the protein-protein interface of JEV- E and its corresponding interactors indicate that the two membrane proteins PLVAP and LRAT packed at domain III region bind more tightly compared to the others.

### Elevated expression of PLVAP and GKN3 in brain membrane fraction of BALB/c mice

**Figure 4:**
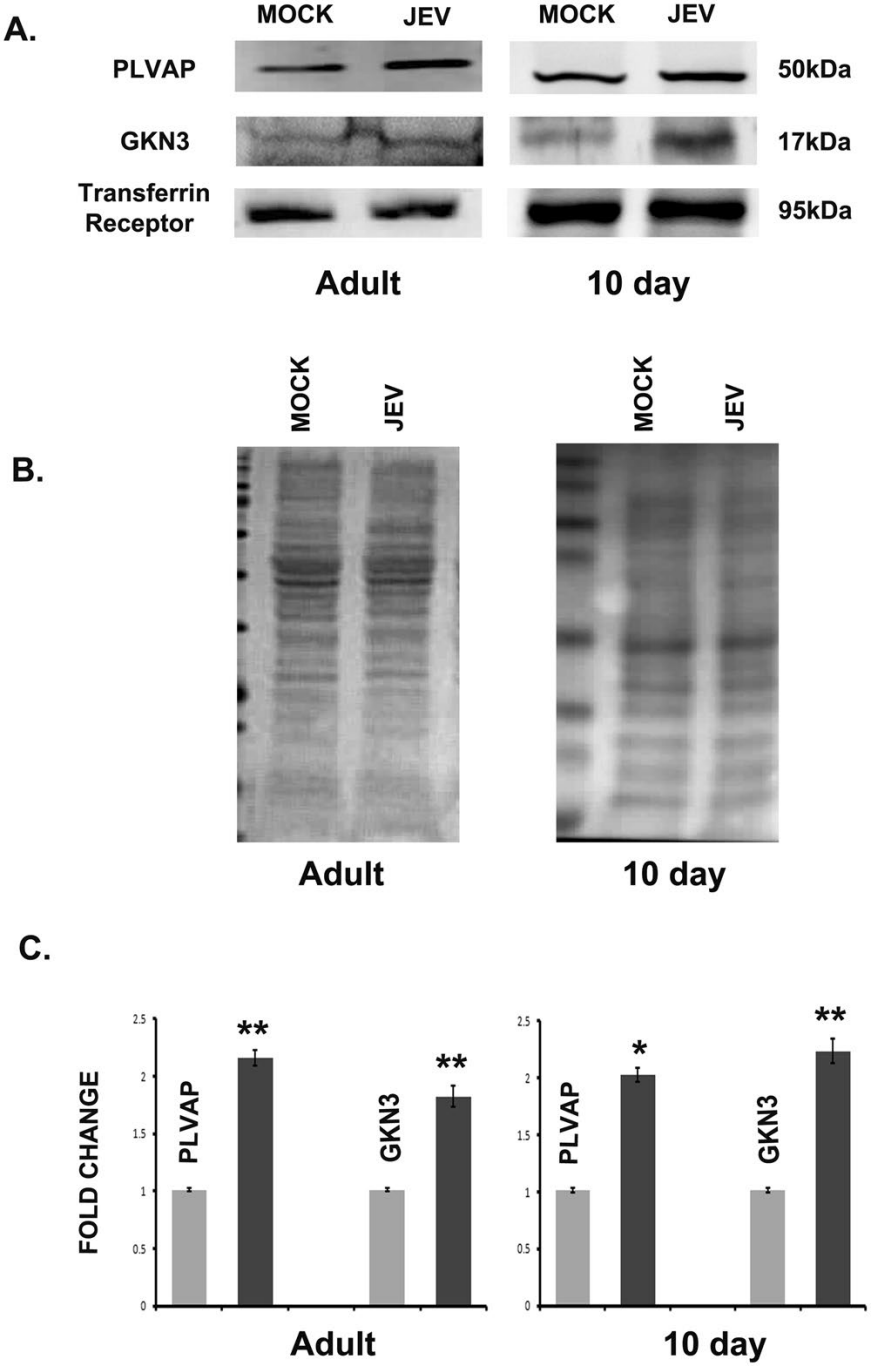
Increased expression of PLVAP and GKN3 in the membrane protein fraction of BALB/c mice brain post JEV infection. (**A**) Immunoblots showing expression of PLVAP and GKN3 in JEV infected brain membrane protein. (**B**) Ponceau staining of membrane proteins in mock and JEV infected adult and 10 day old BALB/c mice. (**C**) Histogram shows significant up-regulation of PLVAP and GKN3 post normalization with transferrin receptor. (*p < 0.5, **p < 0.01). Data is representative of three independent experiments (mean ± SD) by one way analysis of variance (ANOVA) followed by Holm-Sidak *post hoc* test. [Immunoblots were developed from different parts of the same gel after visualizing the ponceau profile].

Membrane proteins, whose mRNA expression was up-regulated in mouse brain post JEV infection, were also validated in the brain membrane fraction by western-blot. Although five membrane proteins were showing increased mRNA expression, we only found enhanced expression of PLVAP and GKN3 in brain membrane fraction of both adult and 10 day old JEV infected mice (Fig. 4A). Ponceau profile of brain membrane proteins indicate equal loading (Fig. 4B). Histograms show densitometric normalization of the result using transferrin receptor as loading control (Fig. 4C). PLVAP and GKN3 were shown to be co-localized with JE virus in mouse brain immuno-histochemistry (Figs S3A, S4B). Their expression in mouse brain was detected in qRT-PCR at day 3 post infection till the appearance of encephalitic symptoms at day 7 (Fig. S3C,D).

### Localization of PLVAP and GKN3 proteins in mouse neuro2a cell membrane is found at earlier time points of JEV infection

**Figure 5:**
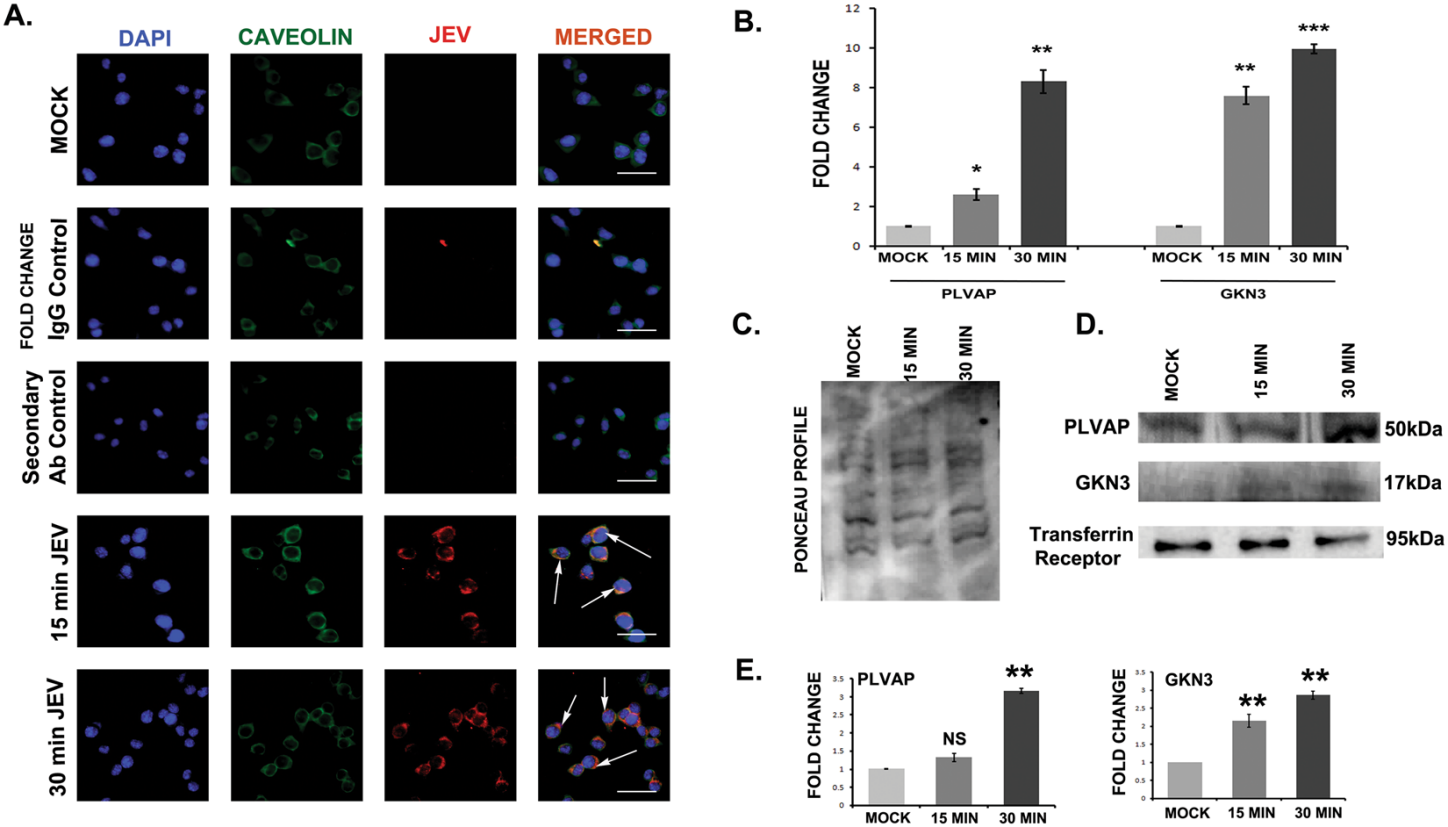
Presence of JEV in neuronal membrane and expression of PLVAP and GKN3 post 15 and 30 min of infection in mouse neuro2a cells. (**A**) JEV co-localizes with Caveolin which is a membrane protein signifying its presence in cell membrane. Scale bar 50 µm, Magnification x20 (**B**) Post 15 and 30 min of viral infection, PLVAP and GKN3 mRNA in neuro2a cells were found to be significantly up-regulated. (**C**) Ponceau image of neuro2a cell membrane fraction post 15 and 30 min of JEV infection. (**D**) Immunoblots showing significant presence of PLVAP and GKN3 proteins in neuro2a cell membrane post 15 and 30 min of viral infection. (*p < 0.5, **p < 0.01,***p < 0.001). Data is representative of three independent experiments (mean ± SD) by one way analysis of variance (ANOVA) followed by Holm-Sidak *post hoc* test. [Immunoblots are performed from different parts of the same gel after visualizing the ponceau profile].

**Figure 6:**
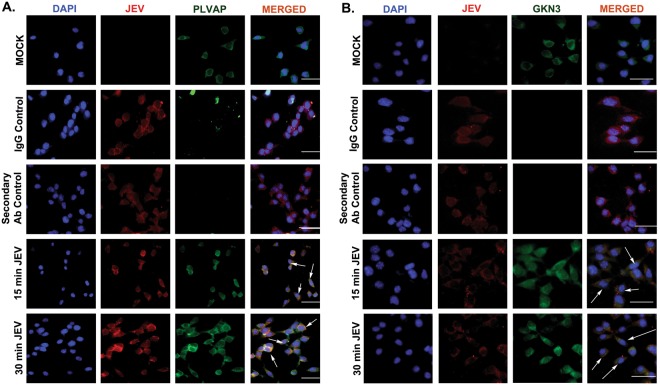
Immunostaining showing co-localization of JEV with PLVAP and GKN3 post 15 and 30 minutes of infection. (**A**) Co-localization of JEV and PLVAP at 15 and 30 min post infection. (**B**) Co-localization of JEV and GKN3 at 15 and 30 min post infection. Scale bar 50 µm, Magnification x20. Data is representative of three independent experiments.

Neuro2a cells were grown till 80% confluency, and then treated with serum free DMEM media. JEV was added to the cells at an MOI of 5 for 15, 30, 45,60,75,90 and 120 minutes respectively. mRNA expression profiles of PLVAP and GKN3 were validated by qRT-PCR and higher expression was found at 15 and 30 min post infection (Fig. S4A). We observed membrane localization of the virus at this early time point. JEV was found to be co-localized with Caveolin which is a known membrane protein (Fig. 5A), suggesting presence of the virus in the cell membrane at this early time point. mRNA expression profiles of PLVAP and GKN3 were validated by qRT-PCR. Both proteins were found to be significantly expressed when compared to mock (Fig. 5B). These receptor proteins were also significantly found to be located in the membrane fraction of neuro2a cells when normalized either with ponceau profile (Fig. 5C) or transferrin receptor expression (Fig. 5D). Neuro2a cells were also fixed with PFA and immunocytochemistry was performed. At both early time points post infection, JEV was found to be co-localized with PLVAP (Fig. 6A) and GKN3 (Fig. 6B). With progressive time points of infection, membrane localization of these receptor proteins increased. Co-IP with purified E-glycoprotein and neuro2a membrane protein confirmed the interaction of PLVAP and GKN3 receptors with JEV E-glycoprotein (Fig. S4B). Similar observations were witnessed in human neuroblastoma cells SH-SY5Y. We validated the expression of both PLVAP and GKN3 in SH-SY5Y cells at 15 and 30 min post JEV infection. No amplification of GKN3 was observed while there was significant up-regulation of PLVAP at 30 min post infection (Fig. S5A). PLVAP was found to be co-localized with JEV at SH-SY5Y membrane post 15 and 30 min viral infection and was elevated at membrane protein fraction also (Fig. S5C,D). Co-IP with purified E-glycoprotein and SH-SY5Y membrane protein confirmed the interaction of PLVAP receptor with JEV E-glycoprotein (Fig. S5E).

### Purified E-glycoprotein treatment elicited expression and membrane localization of PLVAP and GKN3

Mouse neuro2a cells and primary cortical neurons were treated with 20 µg/ml purified and dialyzed E-glycoprotein for 15 and 30 minutes. Thereafter cells were either harvested for RNA isolation or fixed in 4% PFA for immuno-cytochemistry work. E-glycoprotein treatment increased the expression of PLVAP and GKN3 in both neuro2a cells (Fig. S6A) and primary cortical neurons (Fig. S6B). Increased membrane localization of these proteins were validated through immuno- cytochemistry experiments in both neuro2a cells (Figs S7, S8)and in primary cortical neurons (Figs S9, S10). In all cases, these proteins were co-localized with a known membrane protein transferrin receptor.

### *In vitro* modulation in the expression of PLVAP and GKN3 in neuro2a cells can regulate viral replication

**Figure 7:**
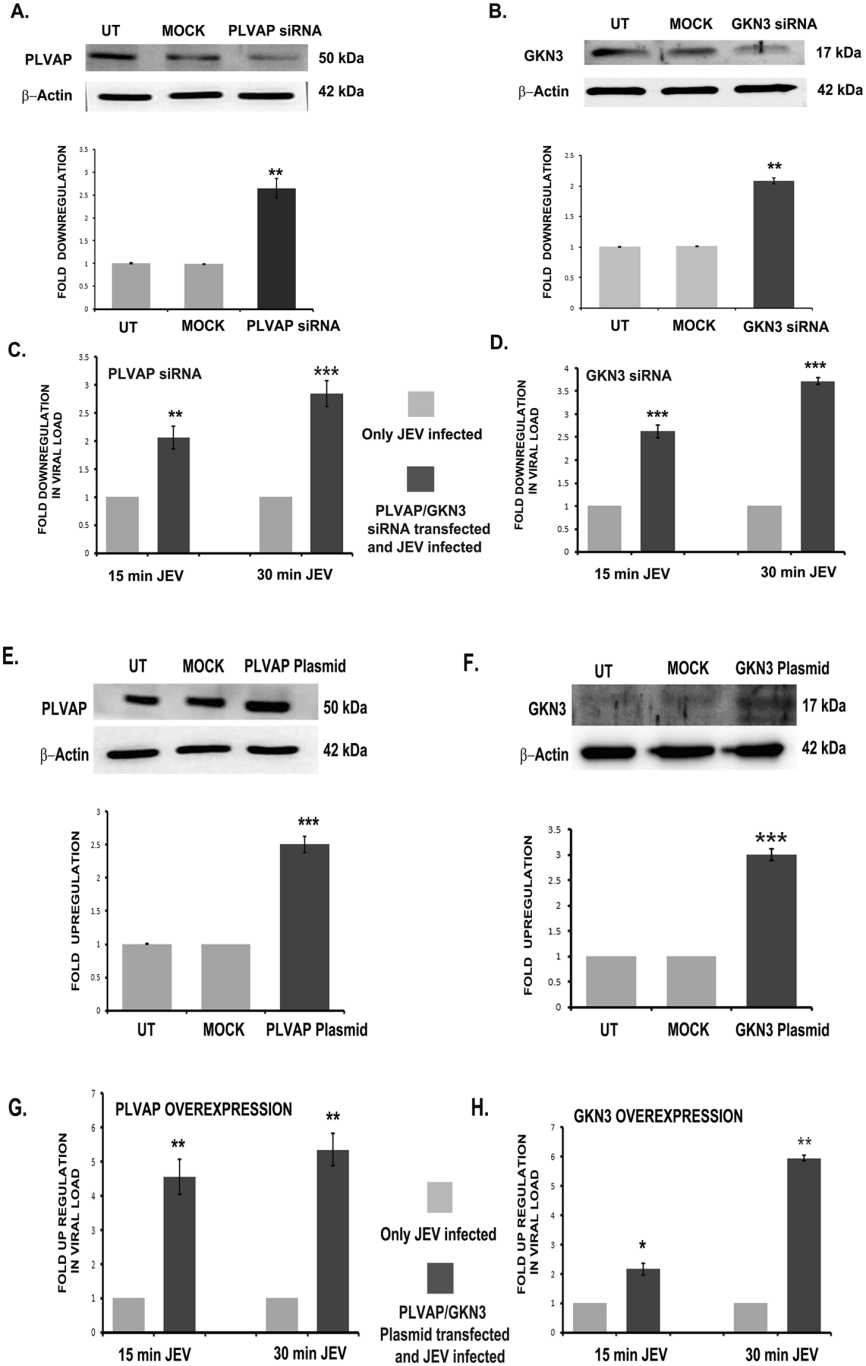
Silencing and over expression of PLVAP and GKN3 by siRNA transfection in neuro2a cells and its effect on viral load. Immunoblots showing significant down-regulation of (**A**) PLVAP and (**B**) GKN3 proteins in neuro2a cells post 48 hours of siRNA transfection, when compared to untransfected and mock (scrambled siRNA). Significant down-regulation in viral load was observed in qRT-PCR after silencing of (**C**) PLVAP and (**D**) GKN3 in neuro2a cells post 15 and 30 minutes of viral infection when compared to only JEV infected cells. Immunoblots showing significant up-regulation of (**E**) PLVAP and (**F**) GKN3 proteins in neuro2a cells post 48 hours of transfection with plasmid, when compared to untransfected and mock (empty vector). Significant up-regulation in viral load was observed in qRT-PCR after over-expressing (**G**) PLVAP and (**H**) GKN3 in neuro2a cells post 15 and 30 minutes of viral infection when compared to only JEV infected cells. (*p < 0.5, **p < 0.01, ***p < 0.001) Data is representative of three independent experiments (mean ± SD) by one way analysis of variance (ANOVA) followed by Holm-Sidak *post hoc* test. [Immunoblots are performed from different parts of the same gel after visualizing the ponceau profile].

Silencing and over-expression of PLAVP and GKN3 were done in neuro2a cells in order to validate their effect on JEV entry. 30 pmol of esiRNA was applied in neuro2a cells for different time points to check its effect on gene silencing. At 48 hrs. post transfection, esiRNA effectively reduced expression of these proteins (Fig. 7A,B) when compared to untransfected cells. Similarly the plasmids of respective proteins enhanced their expression post 48 hrs. of transfection (Fig. 7E,F). After transfection of either esiRNA or plasmids, cells were infected with JEV at an MOI of 5 for 15 and 30 min. To get a comparative study of effective viral replication, only 15 and 30 min JEV infected cells were used as controls. 24 hrs. post infection, in esiRNA treated cells, there was reduction in viral load (Fig. 7C,D) in both the cases of PLVAP and GKN3 silencing. On the other hand, remarkable up-regulation of viral mRNA was found in both PLVAP and GKN3 plasmid treated cells when compared to JEV infected cells (Fig. 7G,H). In another similar set of experiments, esiRNA and plasmid treated cells were used to check expression of JEV NS3 protein (Fig. S11). Plasmid treated cells showed increased levels of NS3 protein at 24 hrs. post JEV treatment of 15 and 30 min (Fig. S11A, S11B) when compared to only infected cells whereas siRNA treated cells showed decreased expression of NS3 protein at 24 hrs. post JEV treatment of 15 and 30 min (Fig. S11C, S11D) when compared to only infected cells. Release of mature virion from the plasmid and esiRNA treated cells post 24 hrs. of infection was monitored by plaque assay in PS cells. Numbers of plaques were found to be increased (PFU/ml) in plasmid transfected cells and decreased in esiRNA treated cells when compared to only virus infected samples (Fig. S12A, S12B). To further confirm the involvement of PLVAP and GKN3 receptors on JEV entry, mouse neuro 2a cells were incubated with different concentrations of PLVAP and GKN3 antibodies and SH-SY5Y cells were treated with only PLVAP antibody. Later on cells were infected with JEV at an MOI of 5 for 15 and 30 minutes followed by thorough washes. After 6 hrs., viral RNA was checked through qRT-PCR and the antibody treated cells showed significant reduction of viral load compared to the un-treated cells (Fig. S13A–D).

### Expression of PLVAP and GKN3 in mouse primary cortical neuron culture

**Figure 8:**
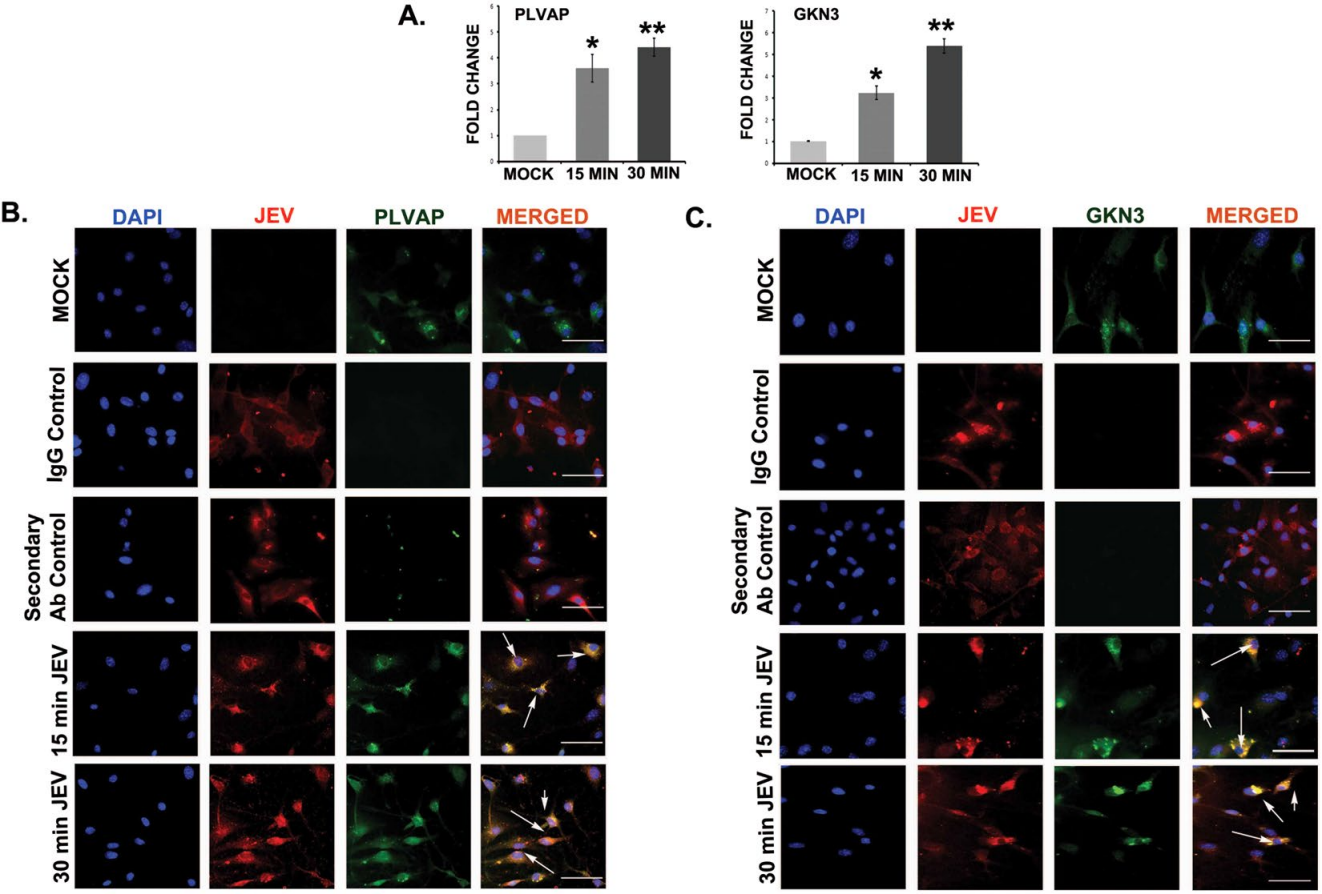
Up-regulation of PLVAP and GKN3 receptors in mouse primary cortical neurons. (**A**) RNA from 15 and 30 min JEV infected cortical neurons were isolated followed by qRT-PCR for PLVAP and GKN3 receptors. Both the receptors were significantly up-regulated. (*p < 0.05, **p < 0.01, mean ± SD) by one way analysis of variance (ANOVA) followed by Holm-Sidak *post hoc* test. (**B**,**C**) PLVAP and GKN3 co-localizes with JEV after 15 and 30 min post infection Scale bar 50 µm, Magnification x20. Data is representative of three independent experiments.

Mouse primary cortical neurons were infected with JEV at an MOI of 5 for 15 and 30 minutes when E glycoprotein interaction with the membrane receptors is supposed to occur. After the treatment, cells were extensively washed with PBS to remove any unbound virus. qRT- PCR was performed with the mock and virus infected samples to check the expression of PLVAP and GKN3 proteins. Both of the proteins showed significant up-regulation at their mRNA level (Fig. 8A). At this particular time point of virus treatment, co-localization of virus with these receptors was clearly seen by immuno- fluorescence (Fig. 8B,C).

### Expression of PLVAP and GKN3 in autopsied human tissues of Japanese encephalitis

Parallel study was conducted in autopsied human tissue of JE-cases to validate the presence of E-glycoprotein interactors previously identified in mouse brain. Presence of JE virus was confirmed in the basal ganglia or frontal cortex autopsy tissue through RT-PCR (Fig. S17A). Age matched control non-JE tissue (accidental cases with minimum trauma to brain) was used for comparison. qRT-PCR experiment showed a significant presence of PLVAP in post-mortem tissue whereas GKN3 was un-detected(Fig. S17B). The study was conducted on three different age groups of JEV infected autopsy cases. In each age group, 2 patient samples were taken.

## Discussion

Viral tropism inside host cell primarily depends on attachment to the cell surface proteins followed by its entry. Therefore, identification of host factors required for virus entry can be extremely important for drug development. Since JEV is a major threat to public health in the Asian subcontinent, identification of its cellular interactors is of immense importance. In the present study we aimed to identify the JEV E-glycoprotein interacting cellular membrane receptors in a BALB/c mice model of viral infection through a pull down based proteomic analysis. We identified two novel neuronal membrane receptors namely PLVAP and GKN3 which play an important role in viral entry.

Previous studies have identified many receptors such as HSP70 and GRP78; playing important role in JE virus entry in *in vitro* systems^11,12,15,17–19^, but our study is the pioneer one identifying JE virus receptors in BALB/c mouse model. Through mass spectrometry, we identified 42 proteins from mouse brain which interact with JEV E-glycoprotein. Differential expression pattern of these proteins after viral infection was analyzed through qRT-PCR experiments in mock infected and JEV infected mouse brain. Amongst the identified, 5 proteins (PLVAP, GKN3, EXOC8, SRC8, LRAT) were found to be up-regulated at mRNA level in both adult and P10 mouse brain post viral infection. Since, bio-informatics based analysis of protein-protein interaction in disease biology is a growing area of research; we wanted to validate the interaction of these membrane proteins with JEV- E glycoprotein by *in-silico* structural modeling approach using ZDOCK and RDOCK programmes. *In-silico* analyses were carefully validated in our experimental *in vivo* and *in vitro* models of JEV infection. Although, *in silico* predictions indicated favorable binding of PLVAP and LRAT with E-glycoprotein; when analyzed in membrane protein fraction of infected animal brain, only PLVAP and GKN3 receptor proteins were found to be up-regulated. These receptor proteins were also co-localized with JEV in mouse brain (Fig. S3). Therefore, we conducted our further validation experiments in these receptor proteins only. PLVAP was found to interact with E glycoprotein domain III whereas; GKN3 interaction is mediated through the domain II (DII). Unlike DIII, DII does not directly take part in membrane protein binding; but its interaction with GKN3 might be advantageous for the virus to gain entry inside host system.

Up-regulation of PLVAP occurs in glioma signaling and it was identified as a Glioma Endothelial Marker (GEM)^20,21^. Increased expression of PLVAP is also an indication of blood brain barrier damage^22^ which in turn is a key event in JEV pathogenesis^23^. In our experiments also, we observed elevated expression of PLVAP in both adult and 10 day old BLAB/c mouse brain post JEV infection. To find out which cell type of brain is responsible for this response, we infected three major cell types of brain namely neuron, astrocytes and microglia *in vitro* in mouse neuro2a, C8-D1A and N9 cell lines. No changes in PLVAP or GKN3 expression were found in N9 and C8D1A cells (Fig. S14), while there was significant up-regulation in PLVAP expression in neuro2a cells post 15 and 30 minutes of JE infection when the E-glycoprotein interaction is supposed to take place with the membrane proteins. At these early time points of infection, PLVAP was shown to be localized at the neuronal membrane. Increased expression of this protein is also observed in the plasma membrane fraction of neuro2a cells post JEV infection.

On the other hand, Gastrokine family of proteins is mainly stomach specific. While GKN1 and GKN2 are functional in human, GKN3 is an inactive pseudo gene^24^ having a premature stop codon^25^. Although it is actively functional in other mammals including mice. GKN3 mRNA and protein are primarily found in mucus neck cells and Brunner’s gland of duodenum^26^. In case of several gastric diseases in mouse, GKN3 has been found to be up-regulated like in cases of fundic atrophy/mucus metaplasia and antral tumorigenesis^27^, profound GKN3 expression is observed. It is also up-regulated in genetically susceptible sheep after nematode infection^28^. However, till date there is no report available showing GKN3 expression in mouse brain post viral challenge. In our studies, we found elevated GKN3 expression in both adult and 10 day old BALB/c mouse brain post JEV infection. This protein is found to be localized in mouse neuro2a cell membrane after 15 and 30 minutes of JEV infection and its expression is also increased in the plasma membrane protein fraction. No change in GKN3 expression was observed in microglia or astrocyte cell lines post early JEV infection. Purified and dialyzed E-glycoprotein treatment on mouse neuro2a cells and primary cortical neurons elevated PLVAP and GKN3 expression levels compared to untreated control, indicating no direct role of glycosylation of the E-protein expressed in bacterial system instead of mammalian system; thus being devoid of post translational modifications.

In support of our observations, we infected human neuronal cells (SH-SY5Y) with JEV for 15 and 30 minutes and checked expression of PLVAP and GKN3 at mRNA level (Fig. S5A). As in case of humans, GKN3 is a pseudo gene, we did not observe its amplification although PLVAP receptor was up-regulated manifold when compared to mock. Immunocytochemistry and Co-IP experiments also support interaction of JE virus with PLVAP receptor in human neuronal cells (Fig. S5C–E). Similar data was obtained in autopsied human tissue samples of JEV infection (Fig. S17). We isolated RNA from the paraffin embedded basal ganglia region of JEV infected patients and found out elevated expression of PLVAP receptor when compared with the age matched control cases.

JEV also infects another major cell population in the brain which are neural stem/progenitor cells and decreases their proliferation^29^. The virus induces endoplasmic reticulum stress and apoptotic response in human neural stem cells^30^. To the best of our knowledge, no previous studies have been done to identify E-glycoprotein interacting membrane proteins in this cell type. We therefore aimed to validate the presence of identified membrane proteins in mouse sub-ventricular zone and human neural stem cells (hNS1). PLVAP was found to be elevated in the membrane fraction of both adult and 10 day old infected BALB/c mice sub-ventricular zone (Fig. S15). There was up-regulation of PLVAP in hNS1 (human neural stem cell line) cells after 15and 30 min of virus infection (Fig. S5B).

Collectively, our data demonstrate a comprehensive role of PLVAP and GKN3 receptor proteins in JEV entry into neurons through interaction with the viral envelop protein. Overexperssion of them facilitates viral entry while their down regulation abrogate the E-glycoprotein binding thus preventing JE infection which is evidenced form decreased viral RNA and protein levels after 24 hrs. post infection. Hence, targeting these membrane receptor proteins in host system using inhibitor molecules can be useful in antiviral research.

## Materials and Methods

### Ethics Statement

Animal Ethics Committee of National Brain Research Centre approved the performed animal experiments (Approval no- NBRC/IAEC/2014/96). Animals were handled with good care according to the guidelines of Committee for the Purpose of Control and Supervision of Experiments on Animals (CPCSEA), Ministry of Environment and Forestry, Government of India. All animal experiments were performed according to the relevant guidelines and regulations.

### Virus propagation

GP78 strain of JE virus was propagated on suckling BALB/c mice. After the appearance of encephalitic symptoms, their brains were harvested and homogenized properly to isolate virus particles. The titre of the isolated virus was calculated through plaque assay in PS cells. The JE virus was handled in a Biosafety Level 2 plus (BSL-2+) laboratory.

### Virus infection in animals

3–4 week and 10 day old BALB/c mice of either sex were divided into two groups. Food and water supply in the cages were ad libitum. Range of body weight of 3–4 week old animals was 12–15 g and for P10 animals it was 4–5 g. No of cage companion in each experiment were variable according to the litter size. Minimum no of animals per experimental group used was at least 10(5 mock, 5 virus infected). In one group of animals of either age group, 3 × 10^5^ pfu of JEV was administered intraperitoneally; while the other group received equal volume of PBS. Both mock infected and virus infected animal brain tissue were collected after the appearance of encephalitic symptoms by perfusion using chilled PBS and stored at −80 °C until used for membrane protein or RNA extraction. In other set of experiments, brains were collected in 4% PFA for immunohistochemistry work. Until cryosectioning brain samples were stored at 4 °C.

### Cell Culture

Mouse neuroblastoma (neuro2a) cells were grown in Dulbecco’s Modified Eagle Medium (DMEM) containing 10% foetal bovine serum. Human neuroblastoma (SHSY-5Y, a kind gift form Steven W. Levison, Rutgers University, New Jersey Medical School, USA) cells were grown in Minimum Essential Medium (MEM) containing 10% foetal bovine serum. Mouse microglial cell N9 (a kind gift from Prof. Maria Pedroso de Lima, Centre for Neuroscience and Cell Biology, University of Coimbra, Portugal) was grown in RPMI-1640 medium supplemented with 10% foetal bovine serum. Mouse Astrocyte cell line C8-D1A (ATCC, USA) was also grown in Dulbecco’s Modified Eagle Medium (DMEM) containing 10% foetal bovine serum. Human neural stem cell line hNS1 a kind gift from Dr. Alberto Martínez-Serrano, Centre of Molecular Biology Severo Ochoa, Autonomous University of Madrid, Spain was cultured according to previously published protocol^30^. Porcine stable kidney cells (PS) were obtained from National Centre for Cell Science, Pune, India and cultured in DMEM media containing 10% foetal bovine serum.

### Virus infection in cells

Neuro2A cells were cultured till 80% confluency followed by serum free media addition. After 2 hours of serum free addition, cells were either mock infected or infected with 5 MOI of JE virus (GP78 strain) for 15 or 30 min. The cells were then harvested for membrane protein or RNA isolation. Viral infection in SHSY-5Y, hNS1, N9 and C8-D1A cells were performed in the same way.

### Cloning and expression of JEV E-glycoprotein

The full-length JEV-E glycoprotein (GP78 strain) gene was PCR amplified using primers JEV-E F (5′-CCGGGATCCATGGGCAATCGTGACTTC-3′) and JEV-E R (5′GCAAGCTTGATGTCAATGGCACATCCAGT-3). The 1280-bp product, corresponding to the full-length mature protein, was purified by gel extraction using a commercial kit (Qiagen, Germany) and was cloned into the pET28a vector (Qiagen, Germany) according to the manufacturer’s instructions. The ligated products were transformed into *Escherichia coli* BL21 (DE3) cells. Plasmids were isolated from 10 randomly selected clones (colonies appeared on agar plate were randomly picked up and grown overnight in 5 ml broth) and were tested for the presence of the insert by size determination on an agarose gel (1%) and PCR amplification of the target gene. Two clones positive for the insert in the correct orientation were subjected to double-pass sequencing to check for possible mismatches using the commercial services (Xcelris Labs, India) employing an automated sequencer. The expression of recombinant protein was induced for 6 hrs. at 25 °C in 250-ml LB medium culture containing 0.2 mM isopropyl-D-thiogalactopyranoside (IPTG). The over-expressed recombinant protein was purified to near-homogeneity by using Ni-NTA Sepharose resin (Qiagen, Germany) according to the manufacturer’s instructions.

### Isolation of plasma membrane protein from cells or mouse brain tissue

Plasma membrane proteins from neuro2a cells or BALB/c mice brain samples were isolated using Plasma Membrane Protein Extraction Kit (Abcam) according to the manufacturer’s instruction. Briefly, cells were collected in ice cold PBS followed by sonication and the tissue was homogenized using homogenization buffer mix provided in the kit. Homogenates were centrifuged and the supernatant thus collected was re-centrifuged at 10000 × g for 30 min. The pellet contained total cellular membrane protein. Plasma membrane protein fraction was separated by repeated phase separations using upper and lower phase solutions provided with the kit following the manufacturer’s instructions. Isolated membrane protein was dissolved in 0.5% Triton X-100 in PBS and subsequently used for further western-blot analyses.

### JEV E-glycoprotein pull down analysis with brain membrane protein

Affinity pull down analysis was carried out as described previously^31^. 1 mg of membrane protein was pre-cleared with Ni-NTA beads to remove non-specific binding to JEV- E-glycoprotein. The pre-cleared lysate was mixed in a micro-centrifuge tube with 5 mg of purified His-tagged JEV E-glycoprotein. The mixture was incubated on a rocking platform at 4 °C for 1 hr, and then 100 μl Ni-NTA bead suspension was added, and samples were incubated another 1 hr at 4 °C with rocking. His tagged E-glycoprotein pulled-down beads were suspended in 8 M Urea and 2% CHAPS to extract the protein complexes and the protein concentration was determined by Bradford’s method.

### 2-DE gel electrophoresis

The pulled down proteins were separated by 2-DE gel electrophoresis using a procedure described earlier^30,32^. 200 µg of the pulled down proteins were precipitated overnight with 10% TCA. The resulting protein pellet was washed twice with chilled acetone and centrifuged at 10000 × g for 10 minutes at 4 °C. The protein pellet was air dried for 5 minutes and resuspended in a buffer containing 7 M Urea, 4% CHAPS and 50 mM DTT and IPG strips of pH range 5-8 and 3-10 were rehydrated overnight for 2DE separation of proteins. The proteins were then focused for 10000 VHr at 20°C on a protean i12^TM^ IEF cell (Bio-Rad, USA). After focusing, the strips were incubated for 10 min, in 2 ml of equilibration buffer I (6 M urea, 30% w/v glycerol, 2% w/v SDS and 1% w/v DTT in 50 mM Tris/HCl buffer, pH 8.8) followed by equilibration buffer II (6 M urea, 30% w/v glycerol, 2% w/v SDS and 4% w/v iodoacetamide in 375 mM Tris/HCl buffer, pH 8.8). After the equilibration steps, the strips were transferred to 12% SDS-PAGE for the second dimension. The proteins were visualized by silver staining or Coomassie Blue R-250 staining. Images of three replicate experiments were captured in LI-COR odyssey image (LI-COR Biosciences, USA). Spots were excised and identified by mass spectrometry at Institute of Life Sciences, Odisha, India.

### Mass spectrometry analysis and database searching

Proteins were identified by mass spectrometry (MS) using an AB Sciex MALDI TOF/TOF 5800 (AB Sciex, CA, USA) at Institute of Life Sciences, Bhubaneswar, after washing and in-gel trypsin digestion of gel spots^33^. All MS and MS/MS spectra were simultaneously submitted to Protein Pilot software version 3.0 (Applied Biosystems) for database searching using Mascot search engine against UniprotKB-Swissprot database containing 544996 sequences with the taxonomy group of *Mus musculus*. Search parameters were as follows: trypsin digestion with one missed cleavage, variable modifications (oxidation of methionine and carbamidomethylation of cysteine), and the peptide mass tolerance of 100 ppm for precursor ion and mass tolerance of ±0.8 Da for fragment ion with +1 charge state. Results obtained from database search were further analyzed. Proteins from *Mus musculus* species with significant Mowse scores and more than one unique peptide were identified and used for further study as shown in Table 1.

### Homology modelling and protein-protein docking

The amino acid sequences of mouse PLVAP, LRAT, GKN3, EXOC8 and crystal structure of SRC8 at 1.65 Å were retrieved from Uniprot and Protein Data Bank (PDB) databases respectively. BLAST (Basic Local Alignment Search Tool) was performed for selection of templates for homology modelling of GKN3, EXOC8 and LRAT against PDB database. Proteins that were used as templates for homology modelling, along with Uniprot IDs, PDB IDs and their identities with the targeted membrane proteins are shown in Table 2. Alignment of template-target sequences were performed by ClustalW. Based on the atomic coordinates of the identical regions of the template structures, the targeted 3D structures of the respective proteins were generated by MODELLER 9v10^34,35^. As no structural analogue of PLVAP was available, ab initio method was used for prediction of 3D structure of PLVAP. ROBETTA server was used for this prediction that uses Rosetta fragment insertion method^36^.

**Table 2:** Sequence identity and modeled region of membrane proteins.

Proteins	PDB ID	Uniprot ID	Template (PDB ID)	Identity (%)	Crystal/Modelled Region (Amino acids)
PLVAP	—	Q91VC4	Ab initio	—	48–438
LRAT	—	Q9JI60	4Q95_A	41.6	41–176
SRC8	3ULR_B	Q60598	—	—	483–546
GKN3	—	Q9D0T7	2YAD_2B	23.8	24–124
EXOC8	—	Q6PGF7	1ZC3_D	38.7	171–279

The crystal structure of ectodomain of Japanese encephalitis virus (JEV) envelope protein (E) at 2.1 Ǻ was retrieved from PDB (PDB ID: 3P54). The envelope protein is composed of separate structural domains. Domain I (DI) consists of 127 residues (1–51, 135–193 and 283–299); domain II (DII) consists of 172 amino acids (52–134, 194–282) and domain III (DIII) consists of a continuous stretch of 100 residues (300–399). The interaction between JEV (PDB ID: 3P54) and its corresponding membrane proteins was studied with protein-protein docking by ZDOCK/RDOCK programmes^37,38^ implemented in Discovery Studio 3. ZDOCK is a rigid body protein docking algorithm that searches rotational space based on Fast Fourier Transformation (FFT) algorithm. Angular step size for rotational sampling was set to 15° which resulted in 3600 poses of each protein-protein complex. The top 2000 poses were retained using ZRANK score and processed by the clustering method. RDOCK algorithm was used for optimization and refinement of docked complexes from each cluster region based on CHARMm-based energy minimization process. The structure with the lowest RDOCK scores (E_RDock) was selected for interaction analysis.

### RNA isolation from mouse brain tissue and neuro2a cells

Both adult (3–4 weeks) and 10 day old mouse brain tissues were homogenised in Trizol reagent (Sigma, USA) followed by addition of chloroform and centrifuged at 12000 rpm for 15 min at 4 °C for phase separation. The aqueous phase was carefully collected and mixed with isopropanol. This was again centrifuged at 12000 rpm for 15 min at 4 °C to get RNA pellet. The pellet was washed in 75% ethanol and air dried. RNA from neuro2a cells were isolated in a similar manner.

### Real time PCR

RNA isolated from both *in vivo* and *in vitro* samples were subjected to cDNA synthesis using advantage RT-PCR Kit (Clontech, Mountain View, USA). The temperature profile for qPCR reaction was 95 °C for 5 min (1 cycle), 95 °C for 30 sec, annealing temperature for 30 sec and 72 °C for 30 sec (45 cycles). The results were normalized using respective human or mouse GAPDH by ∆∆CT method and represented as fold change over mock infected control. Primers used to amplify JEV GP78 were F (5′-3′) TTGACAATCATGGCAAACG; R (5′-3′) CCCAACTTGCGCTGAATAA. Rest of the primer sequences are given in Supplementary Table S10 and S11. Primer sequences were designed at NCBI database through primer blast in the available sequences of our genes of interest.

### RNA isolation from human tissue samples

RNA was isolated from paraffin embedded basal ganglia region of JEV infected human autopsy tissue (CSF positive for JEV–IgM). Age matched non-JEV control samples were accidental cases with least possible trauma to brain. Tissue samples were collected from Human Brain Bank, NIMHANS, Bangalore according to institutional ethics and confidentiality of the subjects. Tissue collection was carried out following institutional rules and guidelines. Consent was obtained from the family members of the subjects. Samples were merged in mineral oil (Sigma, USA) and heated for 2 min at 95 °C to remove paraffin according to published protocol^39^. The process was repeated until paraffin was completely removed. Then the tissue was repeatedly washed in RNAlater solution (Sigma, USA) followed by homogenization in Trizol (Sigma, USA) reagent. The aqueous phase was collected and RNA was precipitated by isopropanol. cDNA preparation and qPCR protocol were similar to that used for mouse brain tissue.

### Mouse primary cortical neuronal culture

2 day old BALB/c mouse pups were decapitated under sterile conditions and cortex was isolated in calcium-magnesium free Tyrode solution under a dissecting microscope. Cortex was digested with Trypsin and DNase enzymes to make single cell suspensions. The suspension was passed through a 127 µm pore size nylon mesh (Sefar) to eliminate cellular debris. The filtrate was centrifuged at 1000 rpm to get the cell pellet. Cells were counted in a hemocytometer and equal number of cells were seeded in poly-D-lysine (Sigma, USA) coated plates in a neurobasal medium containing 2 mM L-glutamine, 1% glucose, 5% FBS, Horse serum and penicillin-streptomycin. After two days, serum was removed from the media to inhibit glial growth and N2 and B27 supplements were added in the media. To eliminate glial presence in the culture, cells were treated with 20 µM arabinoside 1 day prior to viral infection. Neurons were infected with 5 MOI of JEV for 15 and 30 minutes followed by thorough washes with PBS to remove the unbound virus. Thereafter, cells were either immunostained for PLVAP and GKN3 or harvested for RNA isolation and qRT-PCR.

### Immunostaining

Neuro2a cells were either mock infected or infected with JEV at an MOI of 5 for 15 and 30 minutes. The cells were thoroughly washed with PBS and fixed in 4% PFA which was followed by blocking with animal serum in which the secondary antibody is raised in. No permeablization step was performed. The cells were then incubated with primary antibody (PLVAP/GKN3/JEV/Caveolin) at 4 °C overnight [dilution used for each primary antibody was 1:250; sources are mentioned in Supplementary Table S1]. The cells were washed with PBXT (Triton X) and incubated with flurochrome conjugated secondary antibodies (1:500) for 1 hr at room temperature. The cells were then mounted with DAPI (Vector Laboratories, USA) following five washes with PBXT (Triton X) and were subsequently observed under a fluorescence microscope (Zeiss, Germany). Isotype controls (IgG) corresponding to the primary antibody and only secondary antibody controls were included in the experiments to ensure specific antibody binding. Similar protocol was followed in case of immunohistochemistry.

### Immunoblot

Membrane proteins isolated from either mouse brain tissue or neuro2a cells were separated by SDS-PAGE with 30 µg of proteins in each well. After separation, the proteins were transferred on to nitrocellulose membrane and ponceau staining image was captured. The nitrocellulose membrane was incubated with respective primary antibodies (dilutions are provided in Table S1) overnight at 4 °C with gentle shaking. After PBST (Tween 20) washes, blots were incubated with respective secondary antibodies (1:5000, Vector Laboratories, USA) and developed in UVITECH imaging system, Cambridge using ECL reagent(Millipore, CA, USA). Transferrin receptor was used as a loading control for membrane proteins. Alternatively ponceau profile was captured to ensure equal loading.

### Silencing of PLVAP and GKN3 in Neuro2a cells

Neuro2a cells grown up to 60% confluency, were transfected with 30 pmol Endoribonuclease prepared short interfering RNA (esiRNA) against mouse PLVAP and GKN3 (Sigma, USA) with Lipofectamine RNAiMAX (Invitrogen, CA, USA) according to manufacturer’s instruction. Mock transfection was done with scrambled siRNA. Post 48 hrs. of transfection, cells were infected with JEV at an MOI of 5 for 15 and 30 minutes and washed with PBS thoroughly to remove any unbound virus. Then fresh media was added to the cells and 24 hrs. later cells were harvested for determination of viral load checking through qRT-PCR. In another set of experiments, cells were washed thoroughly after 15 or 30 min of viral infection to remove any unbound virus particles and then again kept for 24 hrs. at 37 °C in fresh media for analyzing JEV NS3 protein level through immunoblotting.

### Overexpression of PLVAP and GKN3 in Neuro2a cells

Plasmid constructs of mouse PLVAP and GKN3 genes were obtained from GenScript, USA [PLVAP Clone Id: B96694, GKN3 Clone Id: B96020]. Plasmids were transformed into *E. coli* Top10 strain. Plasmid isolation was performed using Plasmid Midi Kit (Qiagen) following manufacturer’s instructions. The transfection was performed as mentioned above using 1.5 µg of each plasmid with Lipofectamine3000 (Invitrogen, CA, USA) according to manufacturer’s instructions. Mock transfection was done with an empty vector. Post 48 hrs. of transfection, cells were infected with 5 MOI of JEV for 15 and 30 minutes and washed with PBS thoroughly to remove any unbound virus. Then fresh media was added to the cells and 24 hrs. later cells were harvested for viral load checking through qRT-PCR. In another set of experiments, cells were washed thoroughly after 15 or 30 min of viral infection to remove any unbound virus particles and then again kept for 24 hrs. at 37 °C in fresh media for analyzing JEV NS3 protein level through immunoblotting.

### Plaque assay

Release of virion particles from plasmid and esiRNA treated mouse neuro2a cells were monitored in porcine stable (PS) kidney cell monolayer culture. PS cells were seeded in 6 well plates. Culture supernatants collected from transfected and infected cells were subjected to serial dilutions (multiples of 10 folds) and added to PS monolayers and incubated for 2 hrs. at 37 °C. Then the inoculum was removed and PS monolayers were covered with a solution containing 1% low melting agarose, minimum essential media, FBS and antibiotics (penicillin, streptomycin). The plates were kept at 37 °C until the plaques became visible after 72–96 hrs. To count the plaques, PS cells were stained with crystal violet solution after fixation using 10% para-formaldehyde.

### Antibody-blocking experiment

Neuro2a and SH-SY5Y cells were incubated with different concentrations of PLVAP and GKN3 antibodies (N2a: PLVAP and GKN3, SH-SY5Y-only PLVAP) according to the previously published protocol^14^ for 1 hr. at 4 °C. To assess the role of these receptors in viral entry, cells were incubated with 5 MOI of JE virus at 37 °C for 15 and 30 minutes. Then cells were washed with acid citrate buffer (pH 3.0) to remove unbound virus and washed thoroughly with PBS. Then fresh media was added to the cells and they were incubated for 6 hrs. at 37 °C. Cells were then harvested for RNA isolation and qRT-PCR for viral RNA load check.

### Co-IP of E-glycoprotein and membrane receptors

Purification of JEV E-glycoprotein was described earlier. Purified E-glycoprotein was bound to dynabeads containing protein G (Novex, Life Technologies) for 1 hr at 4 °C. Membrane proteins were isolated form Mock infected and JEV infected (15 min and 30 min) neuro2a and SH-SY5Y cells. Equal amount of membrane protein from each samples were then incubated with bead bound E-glycoprotein for 2 hrs. at 4 °C. To ensure specific binding, IgG was incubated with protein G beads and later incubated with 30 min JEV infected membrane protein sample. Elution buffer was added to remove the immunocomplex from the bead. The complex was probed by western-blotting using PLVAP and GKN3 antibodies (stripping and reprobing) as well as E-glycoprotein antibody.

### Purified E-glycoprotein treatment in neuro2a cells and mouse primary cortical neurons

JEV-E glycoprotein was purified using Ni-NTA beads as described previously. Purified proteins were dialyzed before treatment into neuronal cells using Dialysis tubing cellulose membrane (Sigma, USA). Dialyzed protein was quantified and at different concentrations (2.5, 5, 10, 15, 20 µg/ml) it was added to neuro2a cells and primary cortical neurons for 15 and 30 minutes. Same volume of dialysis buffer was added to the cells as a blank control. After treatment cells were washed and used for either RNA isolation or immunostaining.

### Lead Acetate treatment in neuro2a and SH-SY5Y cells

Neuro2a and SH-SY5Y cells were incubated with different concentrations of lead acetate (10–150 µM) for 15 and 30 minutes. Cells were then harvested for RNA isolation and qRT-PCR.

### Statistical Analysis

Data is represented as mean ± SD of three independent experiments (n = 3). Statistical significance was calculated in Sigma Plot (Version 13) using Student’s *t*-test or one way analysis of variance (ANOVA) followed by Holm-Sidak *post hoc* test (for multiple groups). p value < 0.05 was considered to be statistically significant.

## Electronic supplementary material

Supplementary Information

## References

[1] Ghosh D, Basu A (2009). Japanese encephalitis-a pathological and clinical perspective. PLoS neglected tropical diseases.

[2] Campbell GL (2011). Estimated global incidence of Japanese encephalitis: a systematic review. Bulletin of the World Health Organization.

[3] Luca VC, AbiMansour J, Nelson CA, Fremont DH (2012). Crystal structure of the Japanese encephalitis virus envelope protein. Journal of virology.

[4] Crill WD, Roehrig JT (2001). Monoclonal antibodies that bind to domain III of dengue virus E glycoprotein are the most efficient blockers of virus adsorption to Vero cells. Journal of virology.

[5] Rey FA, Heinz FX, Mandl C, Kunz C, Harrison SC (1995). The envelope glycoprotein from tick-borne encephalitis virus at 2 A resolution. Nature.

[6] Acosta EG, Castilla V, Damonte EB (2011). Infectious dengue-1 virus entry into mosquito C6/36 cells. Virus research.

[7] Chu JJ, Leong PW, Ng ML (2006). Analysis of the endocytic pathway mediating the infectious entry of mosquito-borne flavivirus West Nile into Aedes albopictus mosquito (C6/36) cells. Virology.

[8] Nawa M, Takasaki T, Yamada K, Kurane I, Akatsuka T (2003). Interference in Japanese encephalitis virus infection of Vero cells by a cationic amphiphilic drug, chlorpromazine. The Journal of general virology.

[9] Das S, Chakraborty S, Basu A (2010). Critical role of lipid rafts in virus entry and activation of phosphoinositide 3′ kinase/Akt signaling during early stages of Japanese encephalitis virus infection in neural stem/progenitor cells. J Neurochem.

[10] Kalia M, Khasa R, Sharma M, Nain M, Vrati S (2013). Japanese encephalitis virus infects neuronal cells through a clathrin-independent endocytic mechanism. J Virol.

[11] Das S, Laxminarayana SV, Chandra N, Ravi V, Desai A (2009). Heat shock protein 70 on Neuro2a cells is a putative receptor for Japanese encephalitis virus. Virology.

[12] Nain, M. *et al*. GRP78 Is an Important Host Factor for Japanese Encephalitis Virus Entry and Replication in Mammalian Cells. *J Virol* **91**, 10.1128/JVI.02274-16 (2017). 10.1128/JVI.02274-16 PMC5331813 28053106

[13] Nain M, Abdin MZ, Kalia M, Vrati S (2016). Japanese encephalitis virus invasion of cell: allies and alleys. Reviews in medical virology.

[14] Chu JJ, Ng ML (2004). Interaction of West Nile virus with alpha v beta 3 integrin mediates virus entry into cells. The Journal of biological chemistry.

[15] Thongtan T (2012). Characterization of putative Japanese encephalitis virus receptor molecules on microglial cells. J Med Virol.

[16] Kollman PA (2000). Calculating structures and free energies of complex molecules: combining molecular mechanics and continuum models. Accounts of chemical research.

[17] Zhu YZ (2012). Association of heat-shock protein 70 with lipid rafts is required for Japanese encephalitis virus infection in Huh7 cells. J Gen Virol.

[18] Ye J (2013). Heat shock protein 70 is associated with replicase complex of Japanese encephalitis virus and positively regulates viral genome replication. PLoS One.

[19] Wu YP (2011). Japanese encephalitis virus co-opts the ER-stress response protein GRP78 for viral infectivity. Virol J.

[20] Strickland LA (2005). Plasmalemmal vesicle-associated protein (PLVAP) is expressed by tumour endothelium and is upregulated by vascular endothelial growth factor-A (VEGF). The Journal of pathology.

[21] Leenstra S (1993). Endothelial cell marker PAL-E reactivity in brain tumor, developing brain, and brain disease. Cancer.

[22] Shue EH (2008). Plasmalemmal vesicle associated protein-1 (PV-1) is a marker of blood-brain barrier disruption in rodent models. BMC neuroscience.

[23] Mishra MK, Dutta K, Saheb SK, Basu A (2009). Understanding the molecular mechanism of blood-brain barrier damage in an experimental model of Japanese encephalitis: correlation with minocycline administration as a therapeutic agent. Neurochemistry international.

[24] Menheniott TR, Kurklu B, Giraud AS (2013). Gastrokines: stomach-specific proteins with putative homeostatic and tumor suppressor roles. American journal of physiology. Gastrointestinal and liver physiology.

[25] Geahlen JH (2013). Evolution of the human gastrokine locus and confounding factors regarding the pseudogenicity of GKN3. Physiological genomics.

[26] Menheniott TR (2010). A novel gastrokine, Gkn3, marks gastric atrophy and shows evidence of adaptive gene loss in humans. Gastroenterology.

[27] Peterson AJ (2010). Helicobacter pylori infection promotes methylation and silencing of trefoil factor 2, leading to gastric tumor development in mice and humans. Gastroenterology.

[28] Nagaraj SH (2012). Proteomic analysis of the abomasal mucosal response following infection by the nematode, Haemonchus contortus, in genetically resistant and susceptible sheep. Journal of proteomics.

[29] Das S, Basu A (2008). Japanese encephalitis virus infects neural progenitor cells and decreases their proliferation. Journal of neurochemistry.

[30] Mukherjee S (2017). Japanese encephalitis virus induces human neural stem/progenitor cell death by elevating GRP78, PHB and hnRNPC through ERstress. Cell death & disease.

[31] Kumar B, Alam SI, Kumar O (2013). Host response to intravenous injection of epsilon toxin in mouse model: a proteomic view. Proteomics.

[32] Sengupta N, Ghosh S, Vasaikar SV, Gomes J, Basu A (2014). Modulation of neuronal proteome profile in response to Japanese encephalitis virus infection. PloS one.

[33] Swaroop S, Sengupta N, Suryawanshi AR, Adlakha YK, Basu A (2016). HSP60 plays a regulatory role in IL-1beta-induced microglial inflammation via TLR4-p38 MAPK axis. Journal of neuroinflammation.

[34] Marti-Renom MA (2000). Comparative protein structure modeling of genes and genomes. Annual review of biophysics and biomolecular structure.

[35] Sali A, Blundell TL (1993). Comparative protein modelling by satisfaction of spatial restraints. Journal of molecular biology.

[36] Kim DE, Chivian D, Baker D (2004). Protein structure prediction and analysis using the Robetta server. Nucleic acids research.

[37] Li L, Chen R, Weng Z (2003). RDOCK: refinement of rigid-body protein docking predictions. Proteins.

[38] Chen R, Li L, Weng Z (2003). ZDOCK: an initial-stage protein-docking algorithm. Proteins.

[39] Rodriguez-Rigueiro T (2011). A novel procedure for protein extraction from formalin-fixed paraffin-embedded tissues. Proteomics.

