# Supplementary material for: PLVAP and GKN3 Are Two Critical Host Cell Receptors Which Facilitate Japanese Encephalitis Virus Entry Into Neurons
Source: Sci Rep. 2018 Aug 6;8:11784. doi: 10.1038/s41598-018-30054-z (PMC6079088; doi:10.1038/s41598-018-30054-z)

PLVAP and GKN3 Are Two Critical Host Cell Receptors Which Facilitate Japanese Encephalitis Virus Entry Into Neurons

Sriparna Mukherjee[1,2], Nabonita Sengupta[1,#], Ankur Chaudhuri[3], Irshad Akbar[1],Noopur Singh[1], Sibani Chakraborty[3],Amol Ratnakar Suryawanshi[4], Arindam Bhattacharyya[2], Anirban Basu[1].

[1]National Brain Research Centre, Manesar, Haryana-122051, India

[2]Immunology Lab, Department of Zoology, University of Calcutta, 35, Ballygunge Circular Road, Kolkata-700019, India

[3]West Bengal State University, North 24 Parganas, Barasat, Kolkata-700126, India

[4]Clinical Proteomics, Institute of Life Sciences, Bhubaneswar, Odisha-751023, India

[#]Present address: Microbiology and Cell Biology, Indian Institute of Science, CV Raman Avenue, Bangalore, Karnataka-560012, India

#Corresponding Author:

Arindam Bhattacharyya (arindam19@yahoo.com) and/or

Anirban Basu (anirban@nbrc.ac.in) (Orcid id: http://orcid.org/0000-0002-5200-2054)

Supplementary Information:

Table S1: Details of antibodies used in Western-Blot

Antibody	Manufacturer	Molecular Weight (kDa)	Catalogue No	Raised in	Dilution Used
Caveolin	Santa Cruz	22	SC-894	Rabbit	1:500
Transferrin	Zymed	95	13-6890	Mouse	1:2000
LDH	Abcam	36	ab53010	Rabbit	1:1000
Anti-His	Sigma	_	H1029	Mouse	1:2000
PLVAP	Santa Cruz	50	SC-50168	Goat	1:500
GKN3	Cloud Clone	17	PAK528Mu01	Rabbit	1:1000
β-actin	Sigma	42	A3854	Mouse	1:10000
JEV NS3	Gene Tex	70	GTX125868	Rabbit	1:10000

Table S2: Details of kits used in the study

Kit used	Catalogue No.	Provider
Plasma Membrane Protein Extraction Kit	ab65400	Abcam
Plasmid Midi Kit	12143	Qiagen
Advantage RT PCR Kit	639506	Clontech

Table S3: Details of chemicals used in the study

Chemicals used (Abbreviated names)	Catalogue No.	Provider
His-Select Nickel Affinity Gel	P6611	Sigma
Triton -X 100	93443	Sigma
CHAPS	C9426	Sigma
Urea	U5378	Sigma
TRI Reagent	T9424	Sigma
Mineral oil	#163-2129	Bio-Rad
RNA later	R-0901	Sigma
Tween 20	P1379	Sigma
Imidazole	10125	Sigma
Coomassie Blue R-250	C.I 42660	Sigma
Chemiluminescent HRP substrate	WBKLS0100	Millipore
DAPI	H1200	Vector Laboratories
Lipofectamine 3000	L3000015	Invitrogen
Lipofectamine RNAi-Max	13778-075	Invitrogen
N2 supplement	17502-048	Invitrogen
B27 supplement	17504-044	Invitrogen
Poly-D-Lysine	P1024	Sigma
LPS	L6011	Sigma

Table S4: E_RDock score and binding energy of membrane proteins

Membrane Proteins	Best E_RDock (kcal/mol)	Binding Energy (kcal/mol)
PLVAP	-29.27	-162.99
LRAT	-21.31	-156.29
SRC8	-30.47	-87.14
GKN3	-27.85	-88.43
EXOC8	-17.39	-130.06

Table S5: Molecular interaction of JEV-E with PLVAP

Bonds	Protein	Ligand	Interaction Constituents	Distance (Å)
H bond1	SER16	ARG426	SER16:HG - ARG426:O	2.03
H bond2	ASN36	ARG426	ASN36:HD21 - ARG426:O	2.18
H bond3	MET348	PRO404	MET348:HN - PRO404:O	2.02
H bond4	GLY388	GLN435	GLY388:HN - GLN435:OE1	2.38
H bond5	THR349	CYS386	CYS386:SG - THR349:OG1	2.86
H bond6	SER16	ARG426	ARG426:HN - SER16:OG	2.44
H bond7	GLY386	GLN435	GLN435:HE22 - GLY386:O	1.99
Salt bridge	ARG353	ASP377	ARG353:NH1 - ASP377:OD2	4.26

Table S6: Molecular interaction of JEV-E with LRAT

Bonds	Protein	Ligand	Interaction Constituents	Distance (Å)
H bond1	ASN313	ALA106	ASN313:HD22 - ALA106:O	2.41
H bond2	ASN313	THR152	ASN313:HD22 - THR152:OG1	1.97
H bond3	SER149	HIS57	HIS57:HD1 - SER149:O	2.33
H bond4	PRO314	TYR61	TYR61:HH - PRO314:O	1.95
H bond5	GLY318	ASN125	ASN125:HD21 - GLY318:O	2.15
H bond6	ALA399	THR130	THR130:HG1 - ALA399:O	2.25
H bond7	SER401	SER135	SER135:HN - SER401:O	2.15
Pi-cation1	LYS312	PHE58	PHE58 - LYS312:NZ	5.94
Pi-cation2	LYS390	PHE164	PHE164 - LYS390:NZ	6.64
Pi-cation3	HIS397	ARG144	HIS397 -ARG144:NE	5.89

Table S7: Molecular interaction of JEV-E with SRC8

Bonds	Protein	Ligand	Interaction Constituents	Distance(Å)
H bond1	TYR155	ASP512	TYR155:HN - ASP512:OD1	2.49
H bond2	SER156	ASP512	SER156:HN - ASP512:OD2	2.02
H bond3	PHE1	ASP498	ASP498:HN - PHE1:N	2.23
H bond4	THR281	GLN500	GLN500:HE22 - THR281:O	2.10
H bond5	GLU273	ARG533	ARG533:HH22 - GLU273:OE2	2.22
H bond6	GLY5	ASN540	ASN540:HD21 - GLY5:O	2.41
Salt bridge1	ARG44	ASP498	ARG44:NH1 - ASP498:OD2	4.26
Salt bridge2	LYS166	ASP498	LYS166:NZ - ASP498:OD2	4.38
Salt bridge3	LYS209	ASP504	LYS209:NZ - ASP504:OD2	3.42
Salt bridge4	LYS166	ASP510	LYS166:NZ - ASP510:OD2	4.68
Salt bridge5	GLU273	ARG533	GLU273:OE2 - ARG533:NH1	4.48

Table S8: Molecular interaction of JEV-E with GKN3

Bonds	Protein	Ligand	Interaction Constituents	Distance (Å)
H bond1	HIS81	SER56	HIS81:HD1 - SER56:OG	2.34
H bond2	CYS92	SER37	CYS92:HN - SER37:O	2.18
H bond3	LYS93	TRP39	LYS93:HZ1 - TRP39:O	1.98
H bond4	LYS93	ILE30	LYS93:HZ3 - ILE30:O	2.19
H bond5	LYS93	ASP40	LYS93:HZ3 - ASP40:OD1	2.03
H bond6	ARG236	SER37	ARG236:HE - SER37:OG	2.00
H bond7	ARG236	SER37	ARG236:HH21 - SER37:OG	2.47
H bond8	CYS92	ARG31	ARG31:HH21 - CYS92:O	2.46
H bond9	GLU237	ARG31	ARG31:HH22 - GLU237:OE2	2.45
H bond10	GLU79	LYS53	LYS53:HZ1 - GLU79:OE1	2.13
H bond11	GLU79	LYS53	LYS53:HZ1 - GLU79:OE2	2.24
Salt bridge1	GLU237	ARG31	GLU237:OE2 - ARG31:NE	4.78
Salt bridge2	LYS93	ASP40	LYS93:NZ - ASP40:OD2	3.80
Salt bridge3	GLU79	LYS53	GLU79:OE2 - LYS53:NZ	2.72
Pi-cation	PHE242	ARG31	PHE242 - ARG31:NE	3.78

Table S9: Molecular interaction of JEV-E with EXOC8

Bonds	Protein	Ligand	Interaction Constituents	Distance (Å)
H bond1	LYS166	ASP201	LYS166:HZ1 - ASP201:OD2	2.14
H bond2	LYS166	ASP201	LYS166:HZ2 - ASP201:OD1	2.40
H bond3	LYS166	ASP201	LYS166:HZ2 - ASP201:OD2	2.36
H bond4	LYS166	CYS202	LYS166:HZ3 - CYS202:SG	2.28
H bond5	THR317	ARG217	ARG217:HN - THR317:O	2.45
H bond6	LEU4	ASN219	ASN219:HD22 - LEU4:O	2.26
H bond7	ILE46	ARG226	ARG226:HH11 - ILE46:O	2.43
Salt bridge1	LYS138	ASP201	LYS138:NZ - ASP201:OD2	3.75
Salt bridge2	LYS166	ASP201	LYS166:NZ - ASP201:OD2	2.70

Table S10: List of mouse specific primers

**Name of Primer (Mouse)**	**Forward / Reverse**	**Sequence(5’ – 3’)**
**PLVAP**	F	AACTATAATCGCTTCATCGC
	R	GCTTGAAGAGTAAAGCTTCG
**GKN3**	F	CCTTATCATAGTGAGACCTGAG
	R	CAATAAGGCGTCTCATGTTG
**GAPDH**	F	ATGGCAAGTTCAAAGGCACAGTCA
	R	TGG GGGCATCAGCAGAAG G

Table S11: List of human specific primers

Name of Primer (Human)	Forward / Reverse	Sequence(5' – 3')
**PLVAP**	F	GGTCATCTACACGAACAATC
	R	GTCTTCTCCTTGGCTATCTC
**GKN3**	F	TTCGTCCTAACCCCATCCCT
	R	TGTTGTCTCGGATGCTGACC
**GAPDH**	F	GCAAATTCCATGGCACCGT
	R	TCGCCCCACTGATTTTGG

Figure S1:

Immunoblot image showing induction of *E. coli* BL21 (DE3) strain with 0.2 mM IPTG at 25°C.

Significant amount of His-tagged E glycoprotein was found at 6 hrs post induction at 52 kDa when compared to uninduced bacterial cells. Left panel shows position of molecular weight marker and right panel shows the chemilumininescent image .Data is representative of three independent experiments.

Figure S2:

Determination of purity of brain plasma membrane fraction.

Immunoblotting with Caveolin and Lactate dehydrogenase was done to rule out any contamination of cytosolic fraction in the brain plasma membrane proteins. Data is representative of three independent experiments.

Figure S3:

Immunohistochemistry showing localization of PLVAP and GKN3 in mouse brain.

(A,B) Mock and JEV infected brain tissue of 10 day old BALB/c were subjected to immunohistochemistry and PLVAP and GKN3 receptors were found to be co-localized with JEV. (C,D) Progress of JEV infection in mouse brain and pattern of PLVAP and GKN3 receptor expression through qRT-PCR with brain RNA samples. (* $p<0.5$, ** $p<0.01$, *** $p<0.001$). Data is representative of three independent experiments (mean ±SD) by one way analysis of variance (ANOVA) followed by Holm-Sidak *post hoc* test.

Figure S4:

Time point wise PLVAP and GKN3 expression in neuro2a cells post infection and Co-IP of neuro2a membrane proteins with JEV E-glycoprotein.

Neuro2a cells were either mock infected or infected with JEV at MOI 5 for 15, 30, 45,60, 75,90 and 120 minutes. (A) qRT-PCR data showing PLVAP and GKN3 receptor mRNA expression with progressive infection time point with highest up-regulation being at 15 and 30 minutes post infection. (B) Co-IP of neuro2a membrane proteins with purified JEV-E glycoprotein showing interaction of these receptor proteins with viral glycoprotein. . (* $p<0.5$, ** $p<0.01$, *** $p<0.001$). Data is representative of three independent experiments (mean ±SD) by one way analysis of variance (ANOVA) followed by Holm-Sidak *post hoc* test.

Figure S5:

JEV infection induces PLVAP expression in SHSY-5Y (Human neuroblastoma) and hNS1 (Human neural stem cells)cells at early time points.

Cells were either mock infected or infected with JEV for 15 and 30 minutes. Significant up-regulation of PLVAP receptor was found in (A) SHSY-5Y cells post 30 min of JEV infection whereas (B) in hNS1 cells, significant up-regulation of PLVAP was found at both time points when compared to mock. (C)PLVAP was found to be co-localized with JEV at SH-SY5Y membrane at 15 and 30 minutes post infection. (D) PLVAP was found to be elevated in membrane fraction at 30 min post infection. Ponceau profile is indicative of equal loading. (E) Co-IP using purified E-glycoprotein shows its interaction with PLVAP receptor protein. No band in lane 4 is indicative of no nonspecific binding of PLVAP receptor with IgG. (** $p<0.01$). Data is representative of three independent experiments (mean ±SD) by one way analysis of variance (ANOVA) followed by Holm-Sidak *post hoc* test.

Figure S6:

Up-regulation of PLVAP and GKN3 proteins in mouse neuro2a cells and primary cortical neurons upon addition of 20 μg/ml purified E–glycoprotein.

RNA was isolated from both cells after 15 and 30 minutes of purified protein treatment. UT is protein untreated cells and Blank signifies a buffer control in which protein dialysis was performed. (A) qRT-PCR data indicates significant fold change of PLVAP and GKN3 in mouse neuro2a cells after purified protein treatment when compared to both UT and Blank.(*$p<0.05$, ** $p<0.01$, mean ±SD)Data is representative of three independent experiments by one way analysis of variance (ANOVA) followed by Holm-Sidak *post hoc* test. (B) qRT-PCR data of mouse primary cortical neurons also indicate significant fold change of PLVAP and GKN3 when compared to both UT and Blank. (*$p<0.05$, ** $p<0.01$,

mean ±SD).Data is representative of three independent experiments by one way analysis of variance (ANOVA) followed by Holm-Sidak *post hoc* test.

Figure S7:

Purified E-glycoprotein treatment increases membrane localization of PLVAP in neuro2a cells

Mouse neuro2a cells were treated with 20 µg/ml of purified E-glycoprotein for 15 and 30 minutes along with a buffer control. Immunostaining shows co-localization of PLVAP with transferrin receptor signifying its presence in cell membrane. Scale bar 50 µm, magnification x20. Data is representative of three independent experiments.

Figure S8:

Purified E-glycoprotein treatment increases membrane localization of GKN3 in neuro2a cells

Mouse neuro2a cells were treated with 20 µg/ml of purified E-glycoprotein for 15 and 30 minutes along with a buffer control. Immunostaining shows co-localization of GKN3 with transferrin receptor signifying its presence in cell membrane. Scale bar 50 µm, magnification x20.Data is representative of three independent experiments.

Figure S9:

Membrane localization of PLVAP after purified E-glycoprotein treatment in mouse primary cortical neurons.

Mouse primary cortical neuronal cells were treated with 20 µg/ml of purified E-glycoprotein for 15 and 30 minutes along with a buffer control. Immunostaining shows co-localization of

PLVAP with transferrin receptor signifying its presence in cell membrane. Scale bar 50µm, magnification x20. Data is representative of three independent experiments.

Figure S10:

Membrane localization of GKN3 after purified E-glycoprotein treatment in mouse primary cortical neurons.

Mouse primary cortical neuronal cells were treated with 20 µg/ml of purified E-glycoprotein for 15 and 30 minutes along with a buffer control. Immunostaining shows co-localization of GKN3 with transferrin receptor signifying its presence in cell membrane. Scale bar 50µm, magnification x20. Data is representative of three independent experiments.

Figure S11:

Effect of plasmid or siRNA on JEV infection in mouse neuro2a cells

Mouse neuro2a cells were transfected with PLVAP and GKN3 plasmids or siRNAs. 48 hours post transfection; cells were infected with JEV at an MOI of 5 for 15 and 30 minutes. After that cells were washed in PBS to remove unbound virus and kept for 24 hours in fresh media. Immunoblots show significant up-regulation of JEV NS3 protein in (A) PLVAP and GKN3 plasmid treated and JEV infected cells when compared to untransfected and infected cells. (B) Densitometric representation of the Immunoblots. (C) JEV NS3 expression was found to be reduced in PLVAP and GKN3 siRNA transfected cells when compared to untransfected and infected cells. (D) Densitometric representation of the Immunoblots. (* $p<0.5$, ** $p<0.01$) Data is representative of three independent experiments (mean ±SD)by one way analysis of variance (ANOVA) followed by Holm-Sidak *post hoc* test.[NS3 and beta actin immunoblots are generated from same gel].

Figure S12:

Plasmid or siRNA pre-treatment in neuro2a cells changes JEV infection induced plaque formation.

Mouse neuro2a cells were transfected with PLVAP and GKN3 plasmids or siRNAs. 48 hours post transfection; cells were infected with JEV at an MOI of 5 for 15 and 30 minutes. After that cells were washed in PBS to remove unbound virus and kept for 24 hours in fresh media. Media was collected post infection and analysed for the release of effective virus particles through plaque assay in PS cells. (A) Plaque forming units after 15 min of JEV infection in both plasmid and esiRNA treated cells. (B) Plaque forming units after 30 min of JEV infection in both plasmid and esiRNA treated cells (** $p<0.01$). Data is representative of three independent experiments (mean ±SD) by one way analysis of variance (ANOVA) followed by Holm-Sidak *post hoc* test.

Figure S13:

Treatment of PLVAP and GKN3 antibodies to neuro2a and SH-SY5Y cells prior to JEV infection reduces viral load.

Neuro2a cells were treated with different concentrations of both PLVAP and GKN3 antibodies and SH-SY5Y cells were treated with only PLVAP antibody prior to JEV infection. To assess the role of these receptors in viral entry, cells were incubated with 5 MOI of JE virus at 37^0 C for 15 and 30 minutes. Then cells were washed with acid citrate buffer (pH 3.0) to remove unbound virus and washed thoroughly with PBS. Then fresh media was added to the cells and they were incubated for 6 hrs at 37^0C. Cells were then harvested for RNA isolation and qRT-PCR for viral RNA load check. (A,B) 30 -50 µg/ml PLVAP and GKN3 antibody pre- treatment significantly reduced viral load when compared to only JEV

treated samples in neuro2a cells. (C, D) 40-50 µg/ml PLVAP antibody pre- treatment significantly reduced viral load when compared to only JEV treated samples in SH-SY5Y cells. (*p<0.5, **p<0.01, ***p<0.001) Data is representative of three independent experiments (mean ±SD) by one way analysis of variance (ANOVA) followed by Holm-Sidak *post hoc* test.

Figure S14:

PLVAP and GKN3 expression in mouse Astrocyte (C8-D1A) and Microglial cells (N9) at early time points of JEV infection.

Cells were either mock infected or infected with 5 MOI of JEV for 15 and 30 minutes. No significant change in the expression of PLVAP and GKN3 was found in (A) C8-D1A cells (B) N9 cells post 15 and 30 min of JEV infection when compared to mock. Data is representative of three independent experiments (mean ±SD) by one way analysis of variance (ANOVA) followed by Holm-Sidak *post hoc* test.

Figure S15:

Increased expression of PLVAP in the membrane protein fraction of BALB/c mice brain sub-ventricular zone post JEV infection.

(A) Ponceau staining of membrane proteins in mock and JEV infected adult and 10 day old BALB/c mice sub-ventricular zone. (B) Immunoblots showing expression of PLVAP in JEV infected brain sub-ventricular zone membrane protein. (C) Histogram shows significant up-regulation of PLVAP post normalization with Transferrin receptor. (**p<0.01). Data is representative of three independent experiments (mean±SD) by one way analysis of variance

(ANOVA) followed by Holm-Sidak *post hoc* test.[Immunoblots of PLVAP and Transferrin receptor are generated from the same gel after visualizing ponceau profile].

Figure S16:

Lead acetate treatment do not alter expression of PLVAP and GKN3 in neuro2a cells and SH-SY5Y cells

Mouse neuro2a cells and human neuroblastoma cells SH-SY5Y were treated with 10, 25, 50,100 and 150 µM of lead acetate for 15 and 30 minutes. Cells were thoroughly washed with PBS and harvested for RNA isolation. (A) qRT- PCR of PLVAP in neuro2a cells at 15 and 30 min. (B) qRT- PCR of GKN3 in neuro2a cells at 15 and 30 min. (C) qRT- PCR of PLVAP in SH-SY5Y cells at 15 and 30 min. Data is representative of three independent experiments by one way analysis of variance (ANOVA) followed by Holm-Sidak *post hoc* test.

Figure S17:

Presence of PLVAP protein in autopsied human Japanese encephalitis cases.

(A)Presence of virus was analyzed in the basal ganglia region of non JE and JEV infected human autopsy samples by PCR using JEV specific primer. PCR product was visualized in agarose gel. (B) Presence of PVAP protein was checked by qRT-PCR in the JE positive autopsy samples. PLVAP was found to be up-regulated in JE affected basal ganglia region. (**$p<0.01$). Data is representative of three independent experiments (mean±SD) by Student’s t test.

Figure S18:

Schematic diagram indicating the study design.

JEV E-glycoprotein interacts with host cell membrane proteins. Through a proteomic approach with pull down proteins of mouse brain membrane and E-glycoprotein, we have identified PLVAP and GKN3 proteins as viral E-glycoprotein interactors present on neuronal membrane. Down-regulation and up-regulation of these proteins decrease and increase viral load in neurons. Therefore, we propose them as two critical host cell factors governing viral entry.[The schematic is prepared by the authors themselves and hereby declare that it is not copied from any previously published article].

Figure S1:

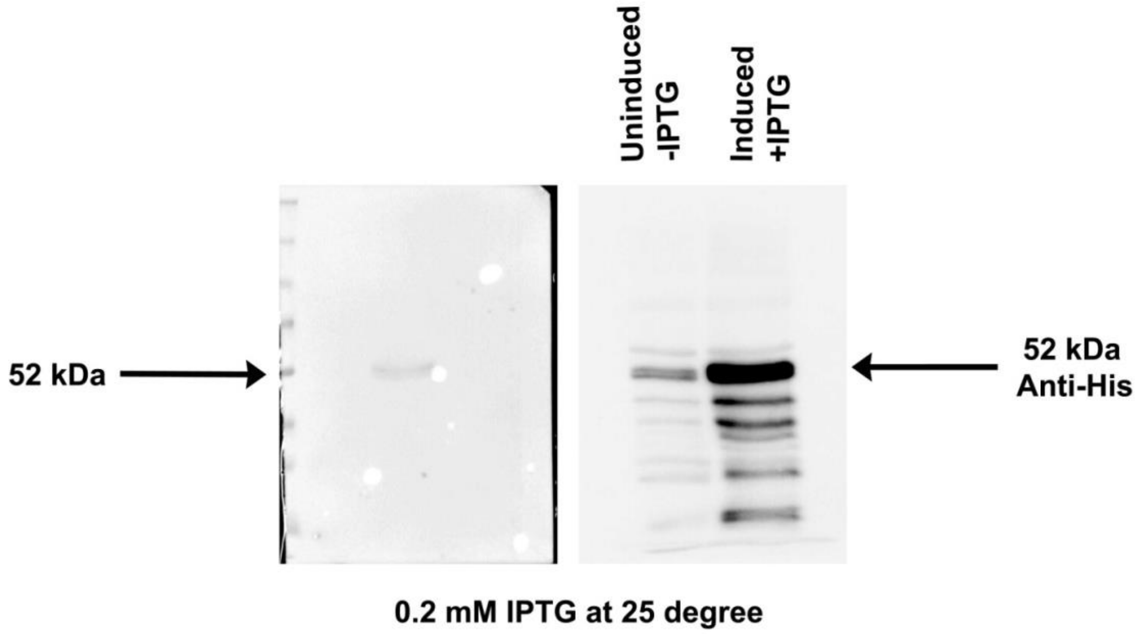

Figure S2:

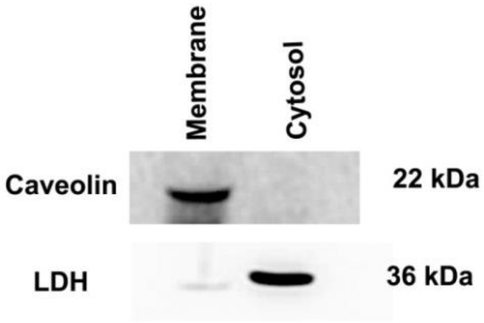

Figure S3:

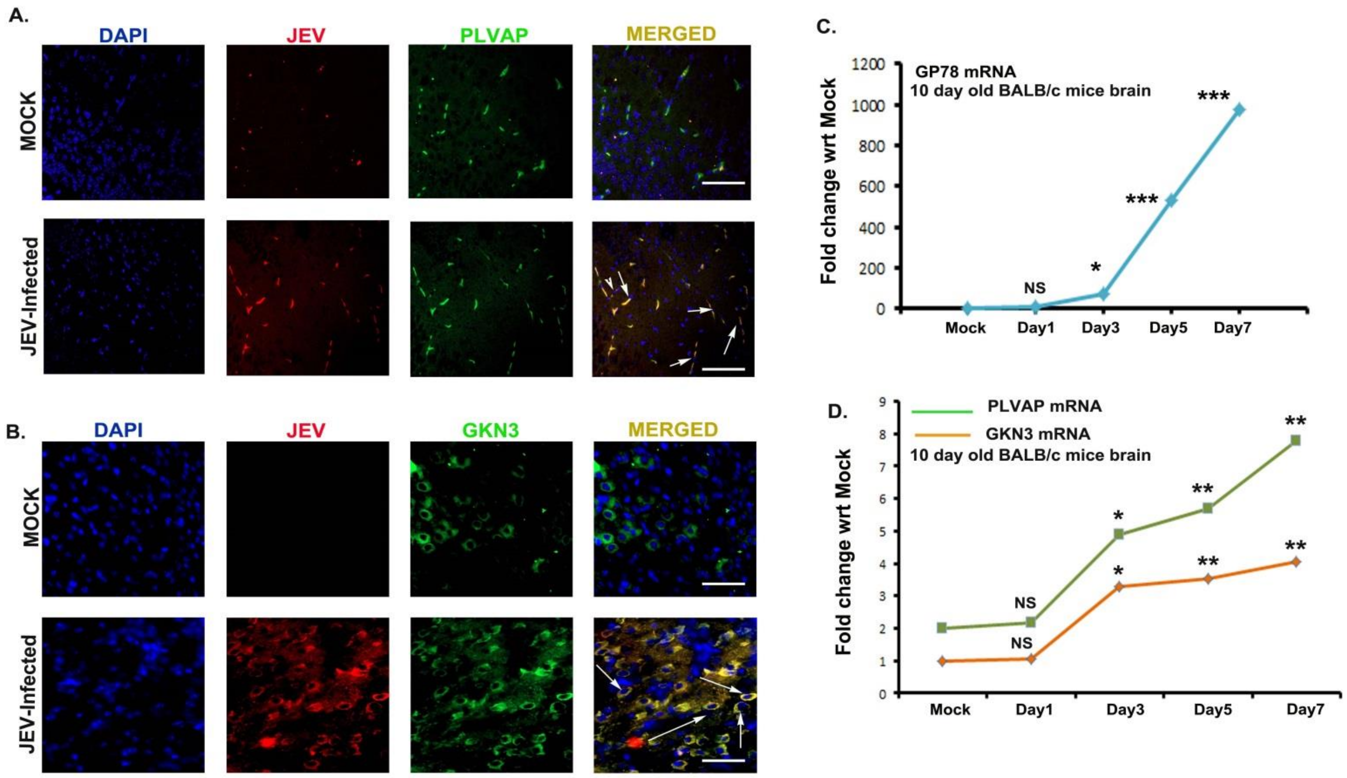

Figure S4:

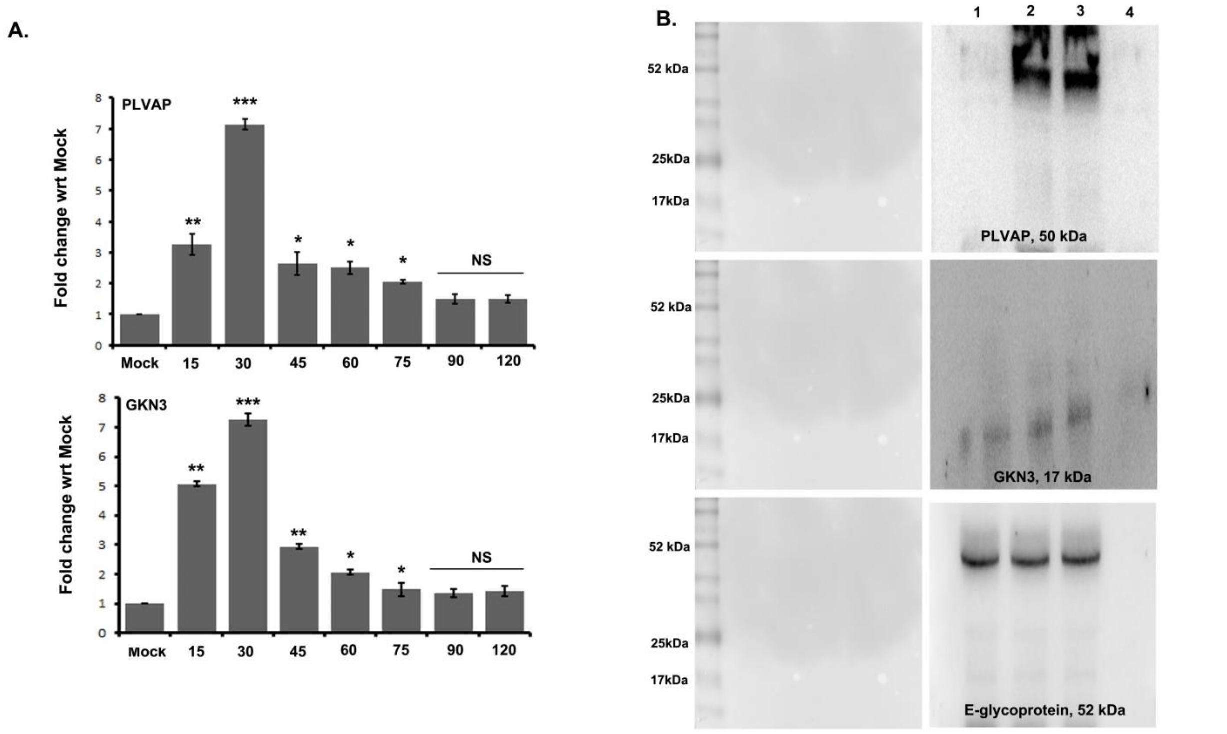

IP: E-glycoprotein in lane 1-3, IgG in lane 4 ;1: Mock, 2:JEV15 min, 3,4: JEV30 min

Figure S5:

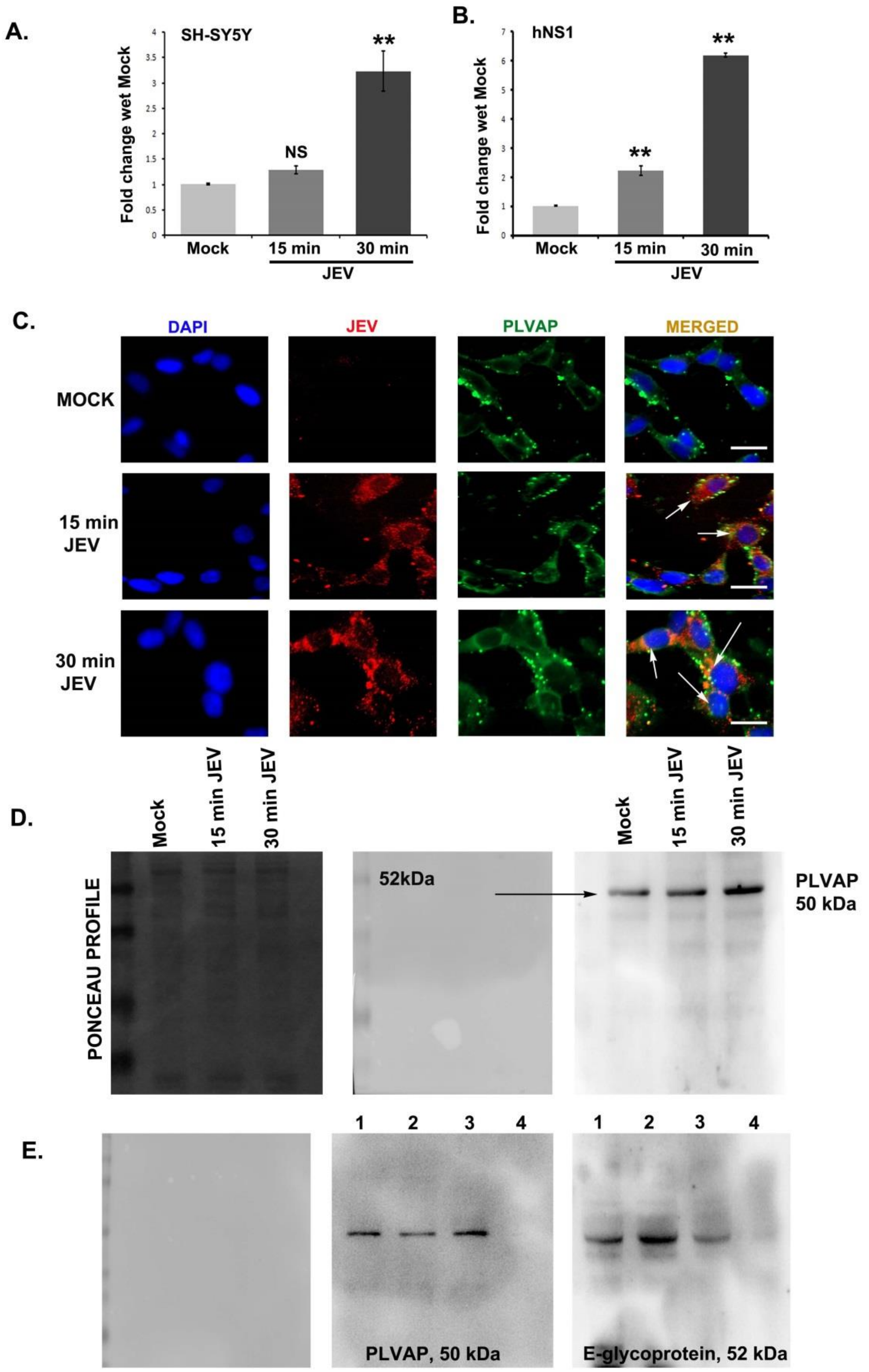

IP: E-glycoprotein in lane 1-3, IgG in lane 4, 1: Mock, 2:JEV15 min, 3,4: JEV30 min

Figure S6:

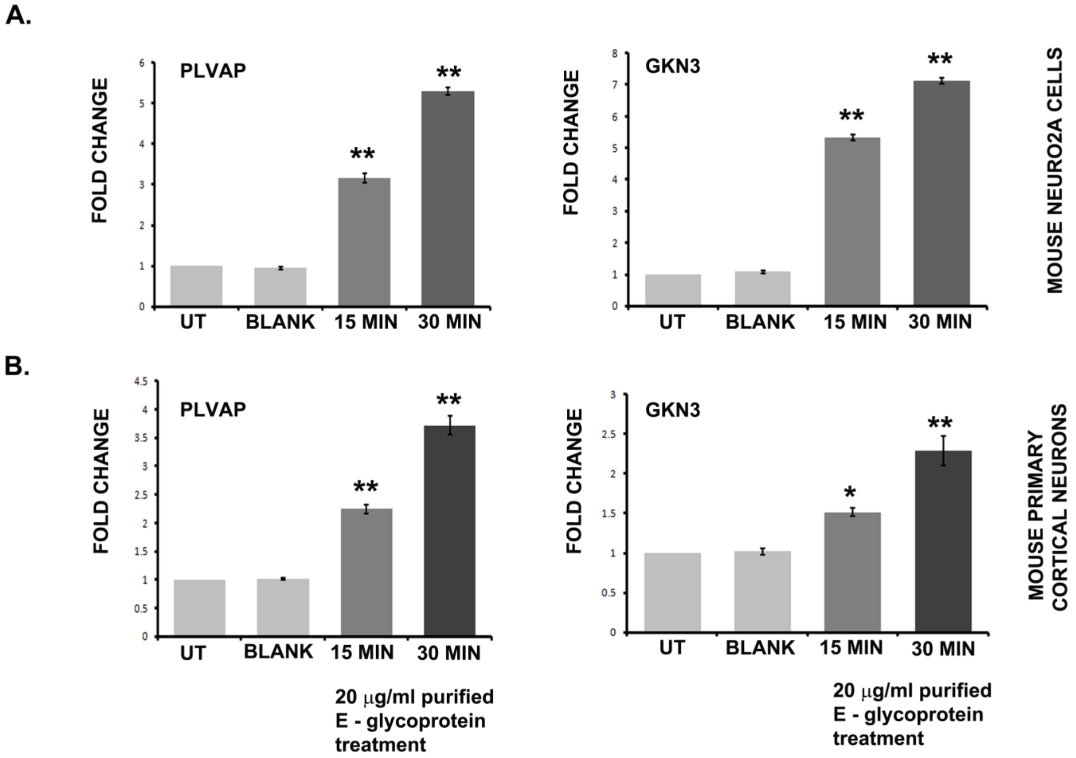
A.
PLVAP
FOLD CHANGE
UT
BLANK
15 MIN
30 MIN
GKN3
FOLD CHANGE
UT
BLANK
15 MIN
30 MIN
MOUSE NEURO2A CELLS
B.
PLVAP
FOLD CHANGE
UT
BLANK
15 MIN
30 MIN
20 μg/ml purified E - glycoprotein treatment
GKN3
FOLD CHANGE
UT
BLANK
15 MIN
30 MIN
20 μg/ml purified E - glycoprotein treatment
MOUSE PRIMARY CORTICAL NEURONS

Figure S7:

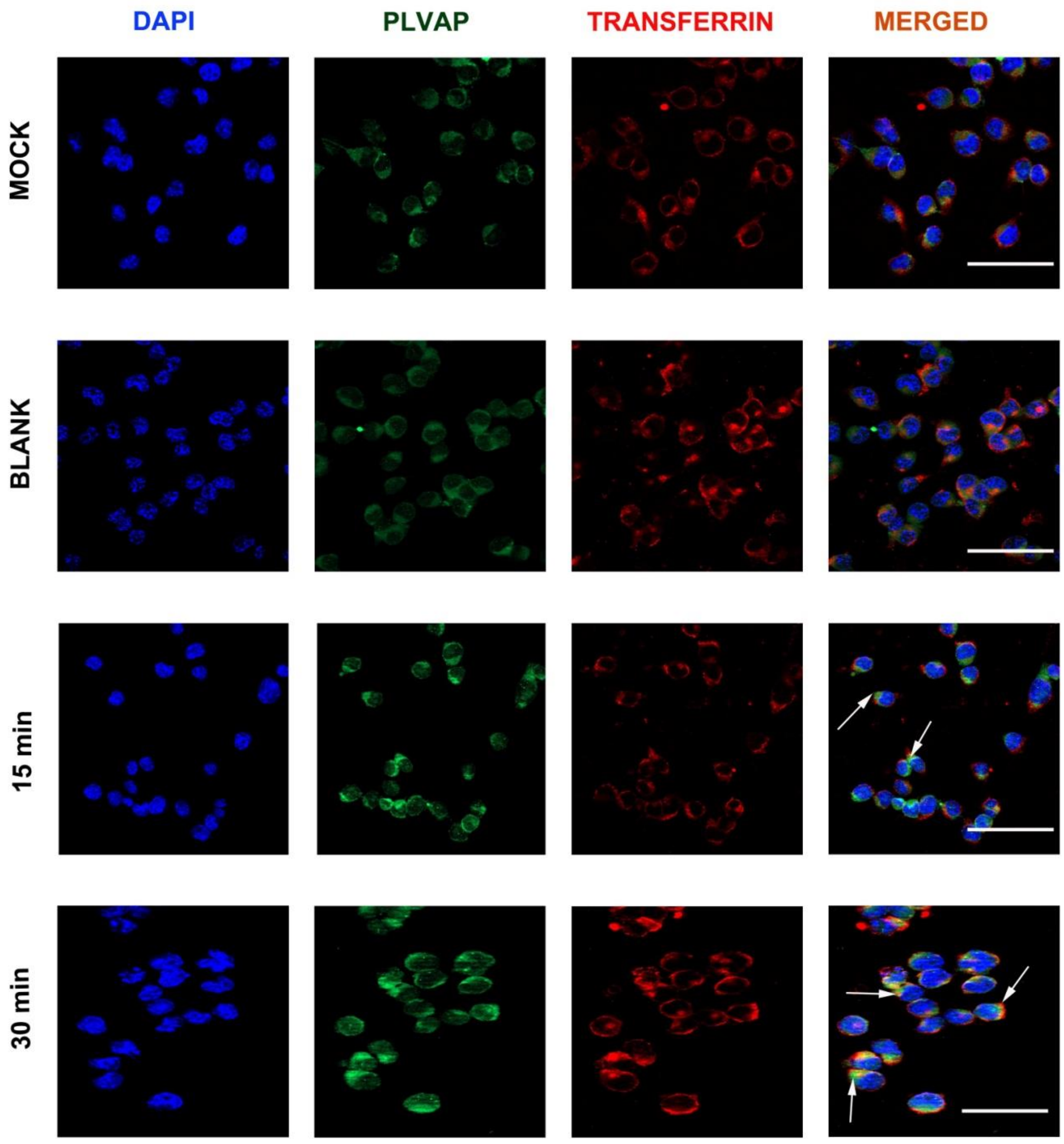

20 μg/ml purified E - glycoprotein treatment for 15 and 30 minutes

Figure S8:

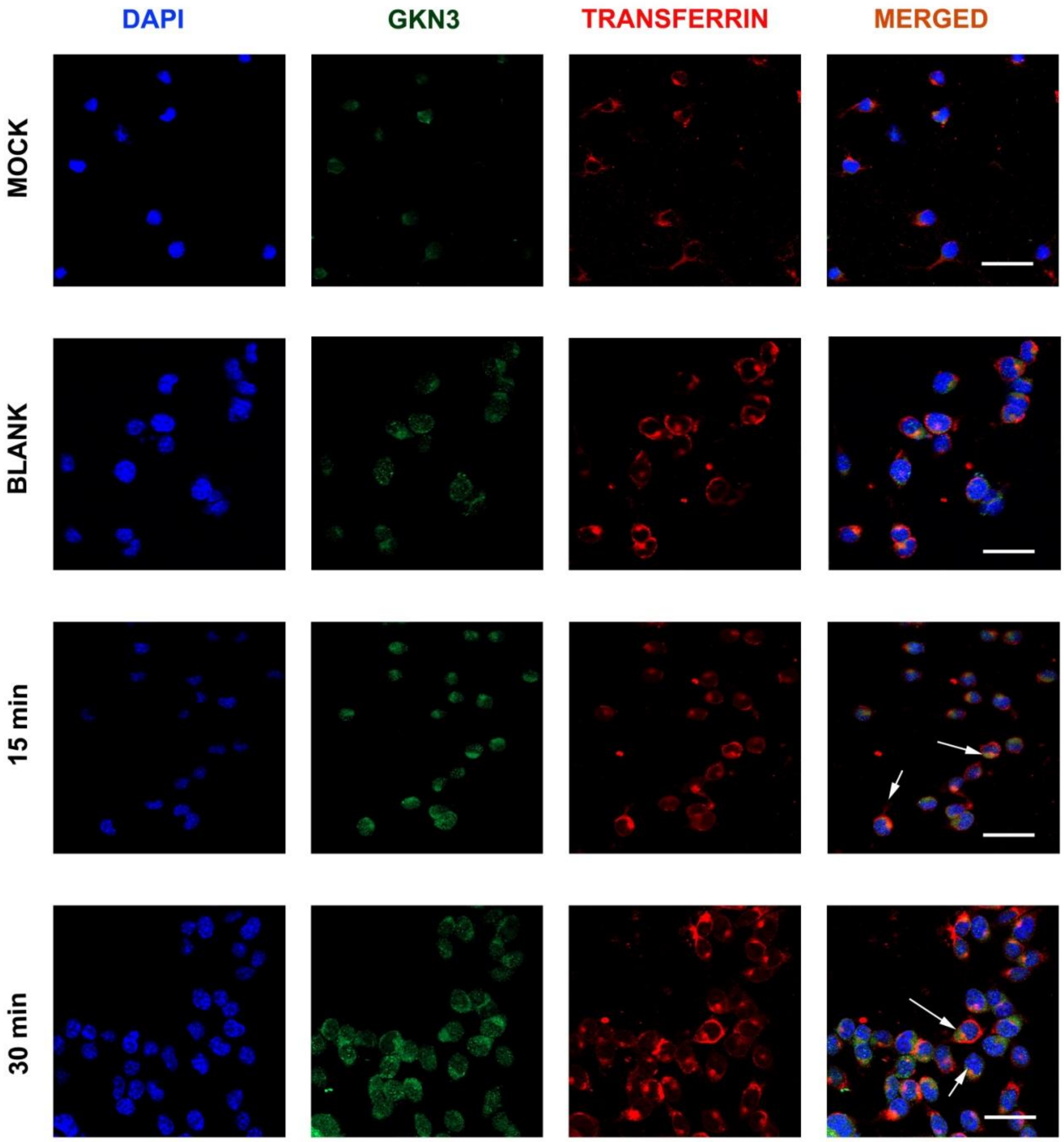

20 µg/ml purified E - glycoprotein treatment for 15 and 30 minutes

314 **Figure S9:**

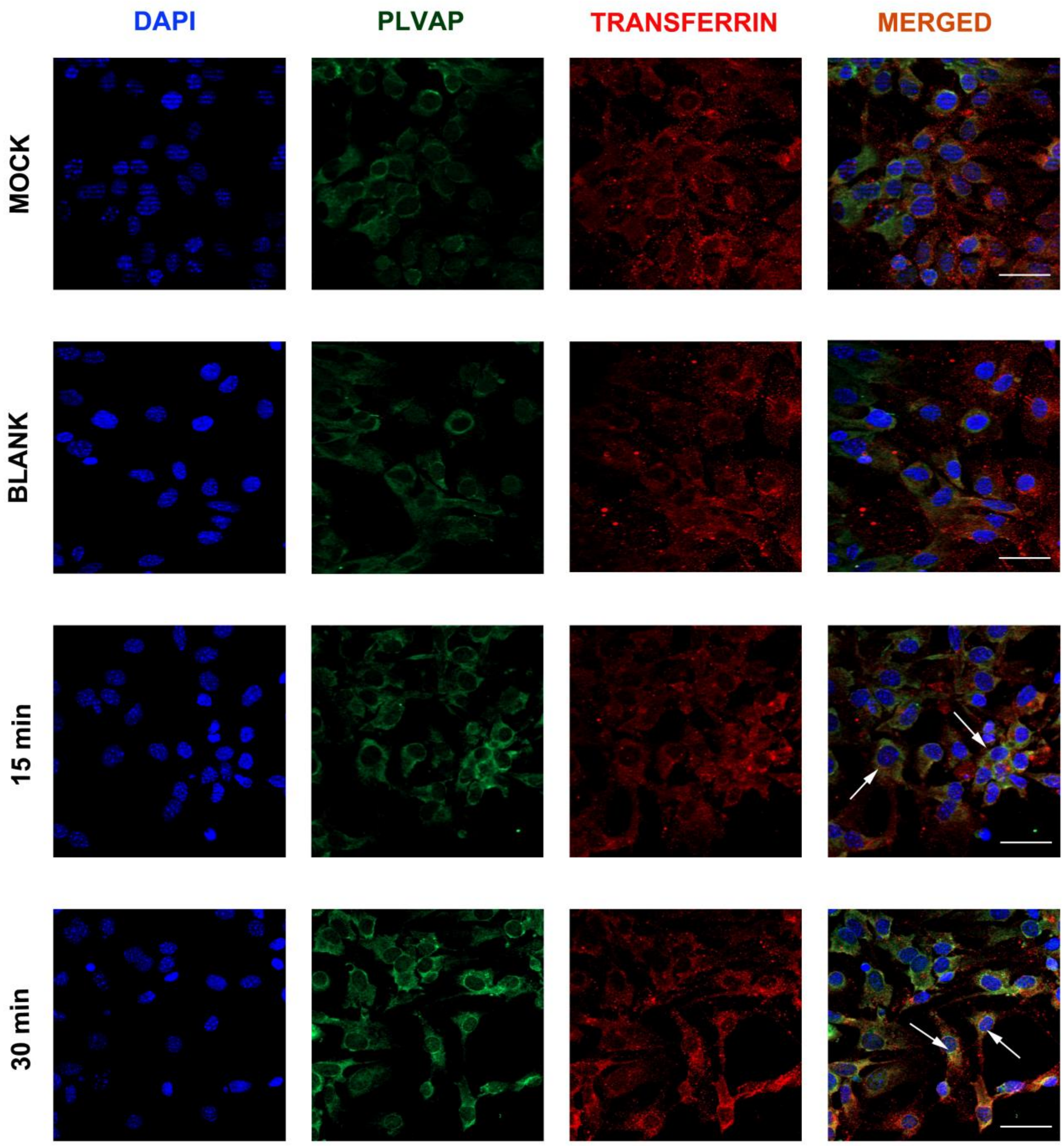

20 μg/ml purified E - glycoprotein treatment for 15 and 30 minutes

315

316 **Figure S10:**

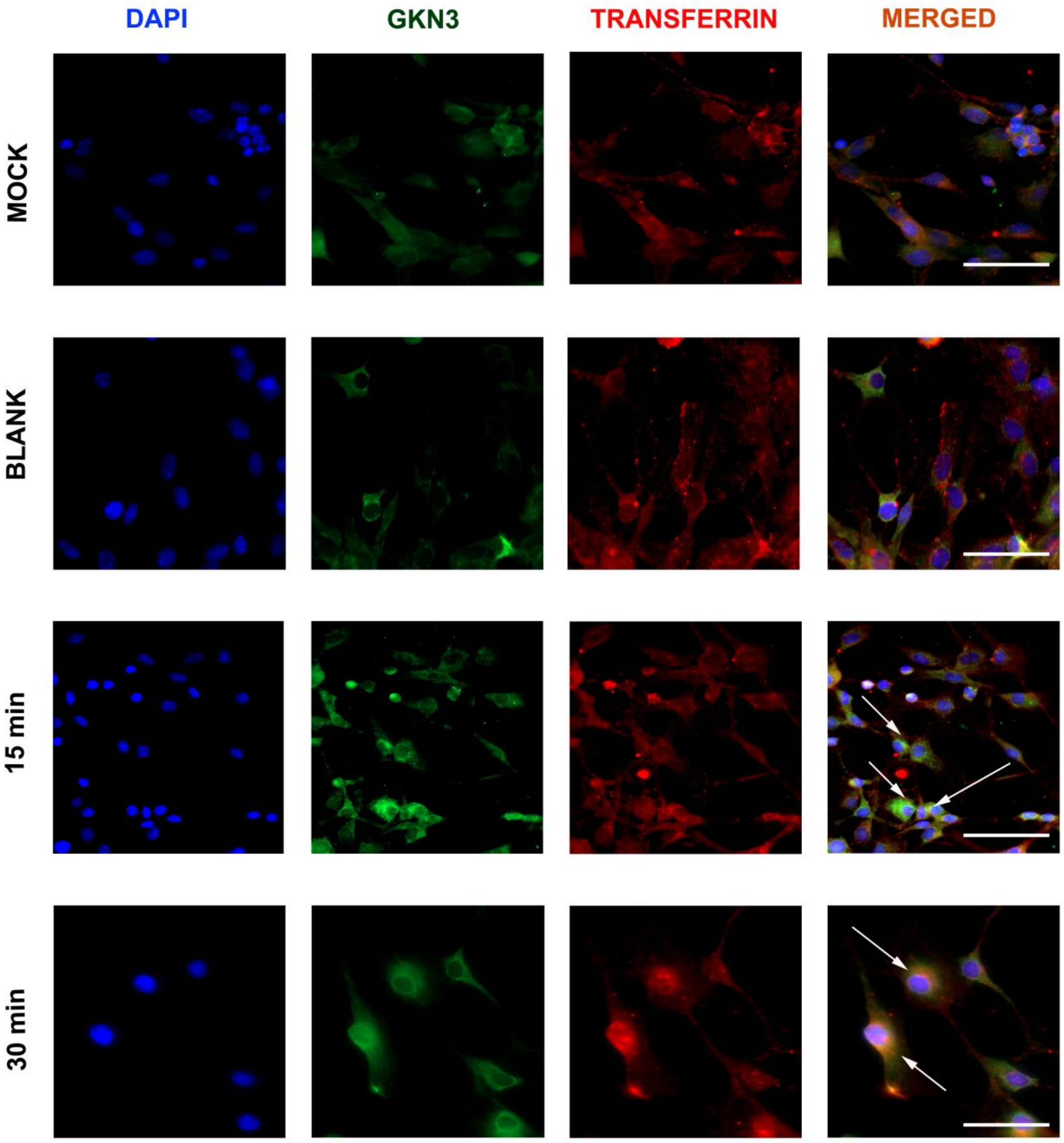

20 μg/ml purified E - glycoprotein treatment for 15 and 30 minutes

317

318 **Figure S11:**

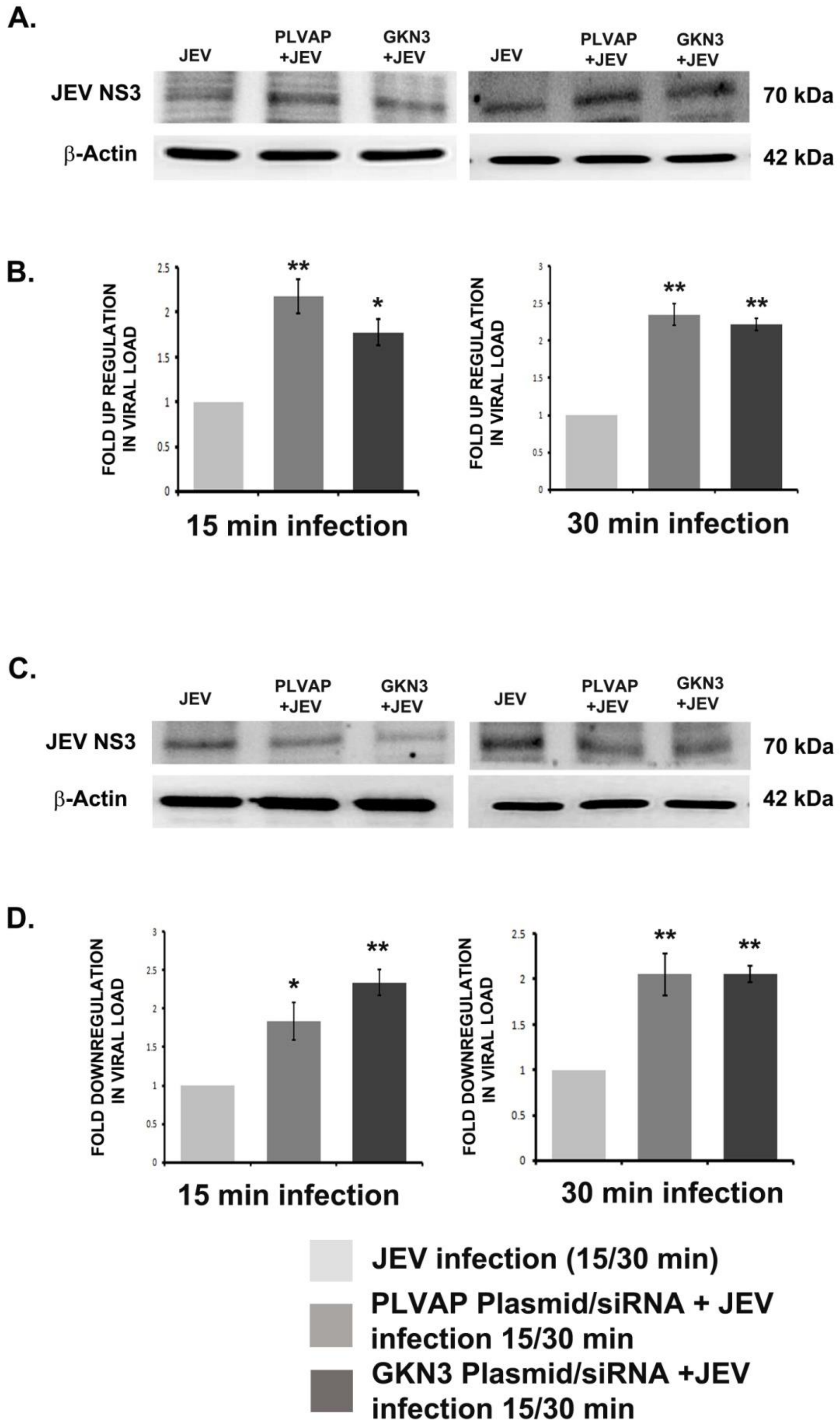

319

Figure S12:

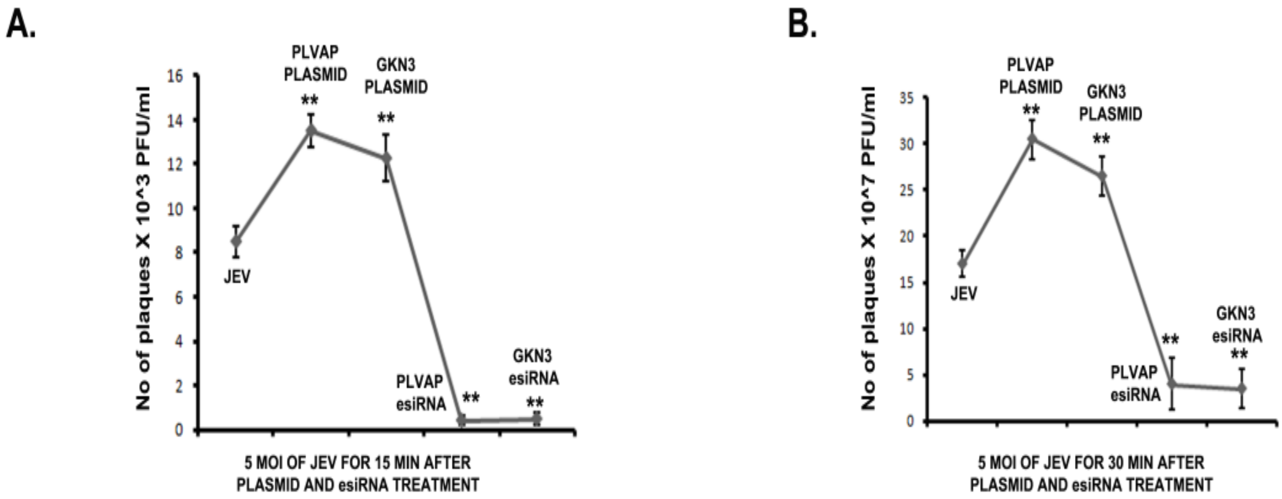

Figure S13:

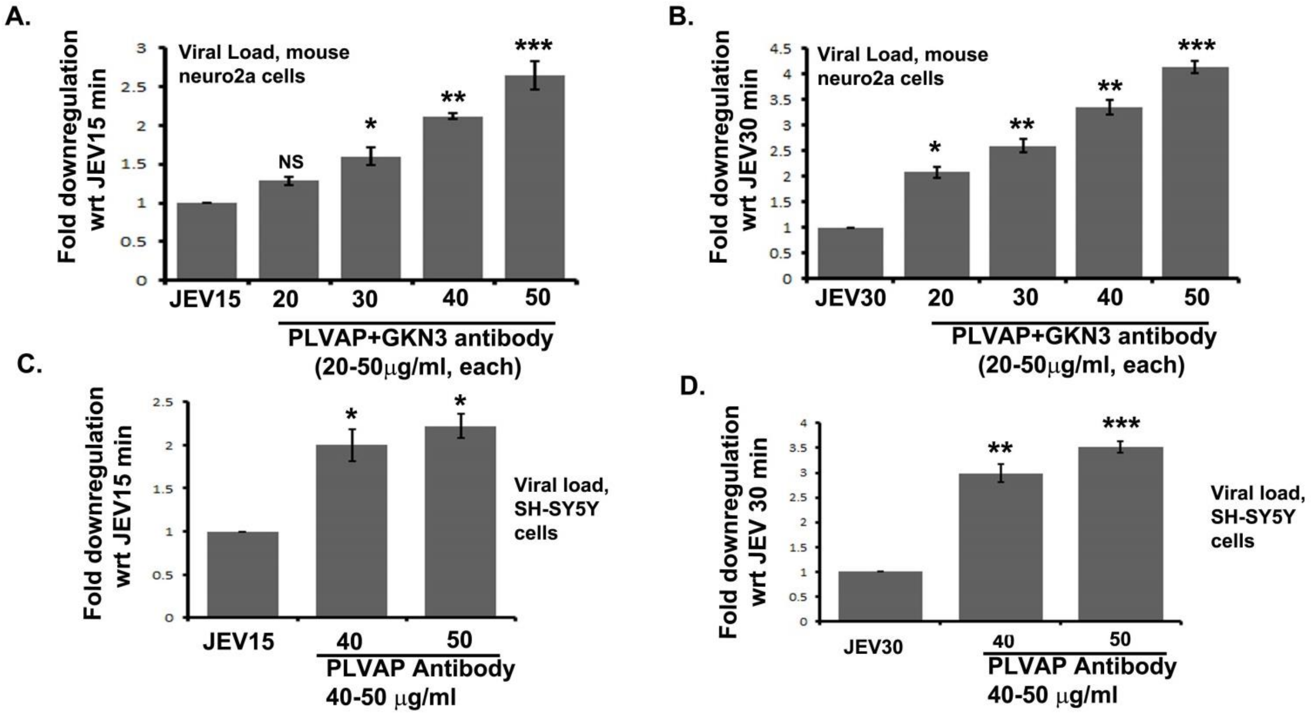

Figure S14:

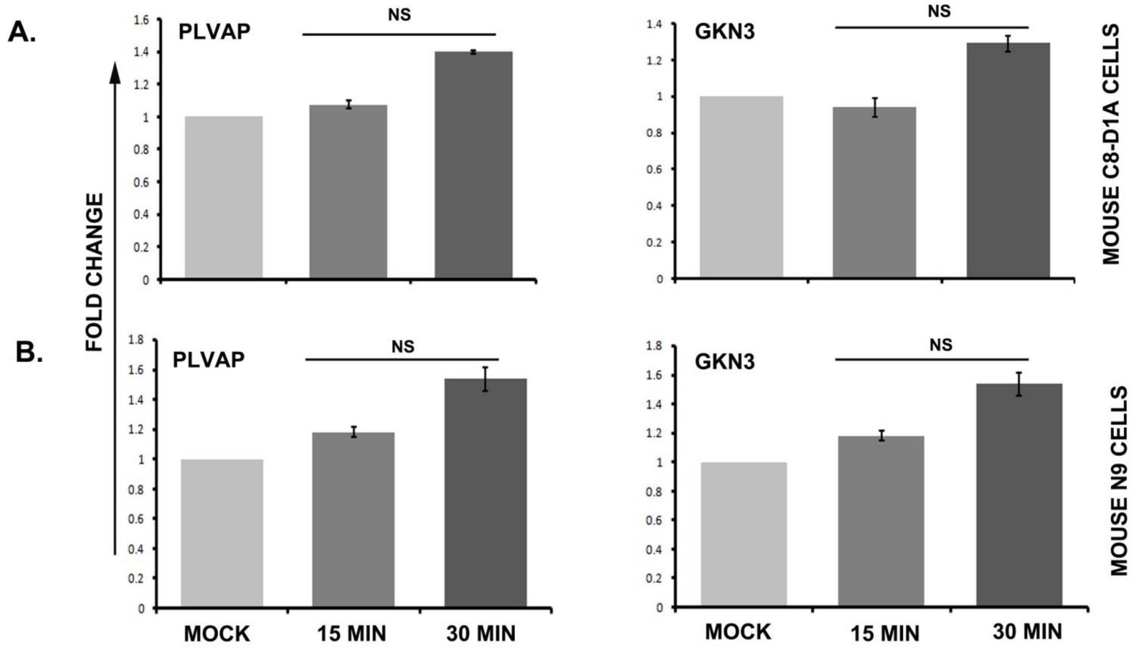
A.
B.
FOLD CHANGE
PLVAP
GKN3
NS
MOUSE C8-D1A CELLS
MOUSE N9 CELLS
MOCK
15 MIN
30 MIN

Figure S15:

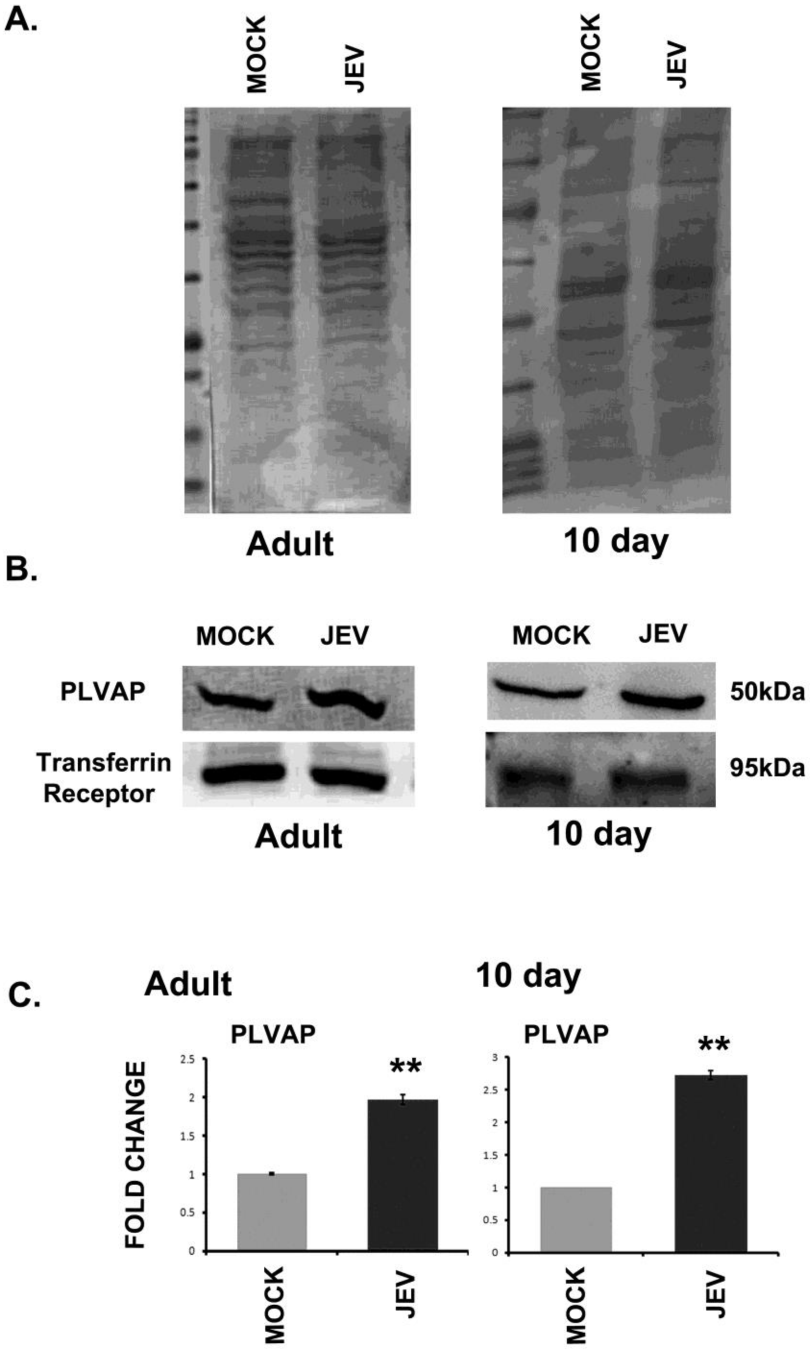
A.
MOCK
JEV
MOCK
JEV
Adult
10 day
B.
MOCK
JEV
MOCK
JEV
PLVAP
Transferrin Receptor
50kDa
95kDa
Adult
10 day
C.
Adult
10 day
PLVAP
PLVAP
FOLD CHANGE
**
**
MOCK
JEV
MOCK
JEV

Figure S16:

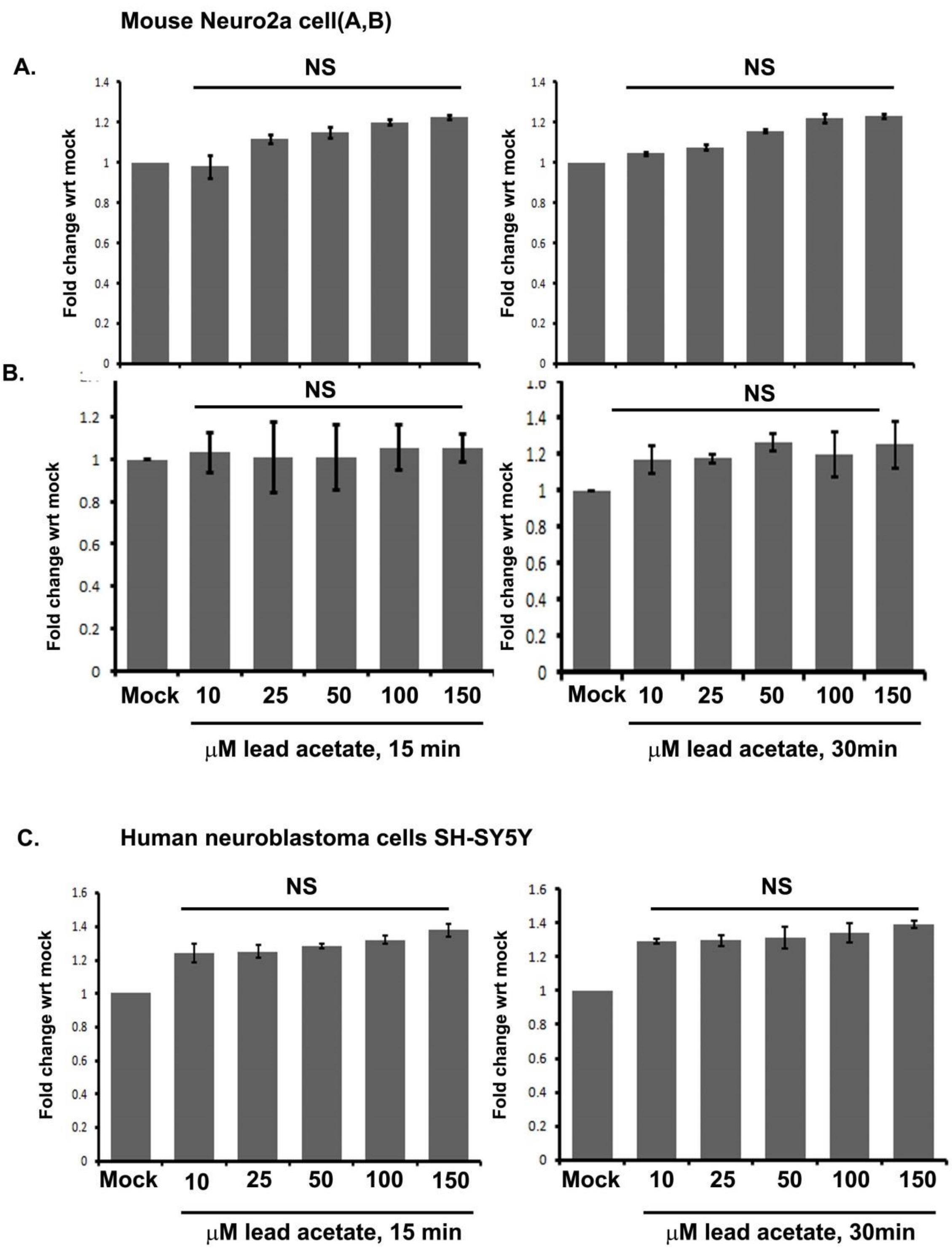
Mouse Neuro2a cell(A,B)
A.
NS
NS
Fold change wrt mock
1.4
1.2
1
0.8
0.6
0.4
0.2
0
B.
NS
NS
1.2
1.4
Mock
10
25
50
100
150
μM lead acetate, 15 min
μM lead acetate, 30min
C.
Human neuroblastoma cells SH-SY5Y
NS
NS
1.6
Fold change wrt mock
Mock
10
25
50
100
150
μM lead acetate, 15 min
μM lead acetate, 30min

Figure S17:

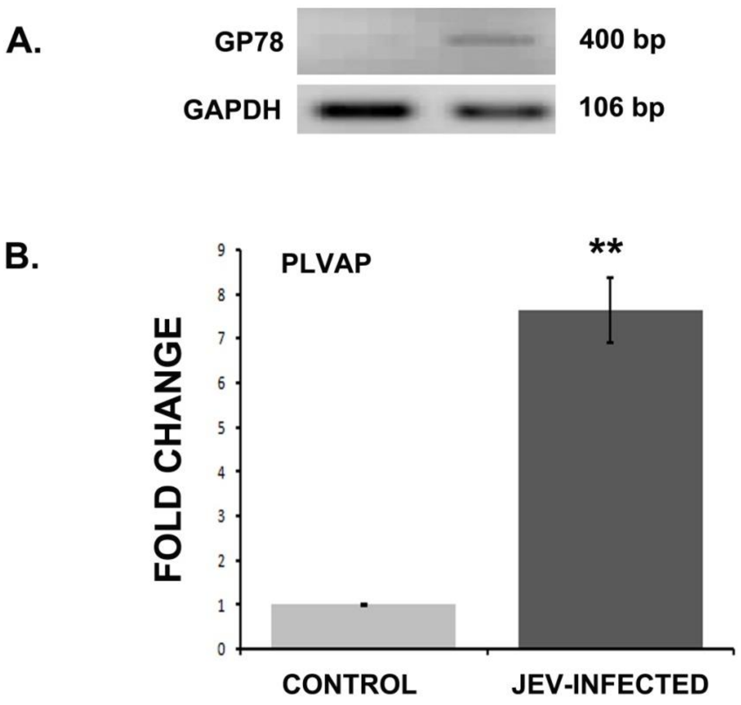

Figure S18:

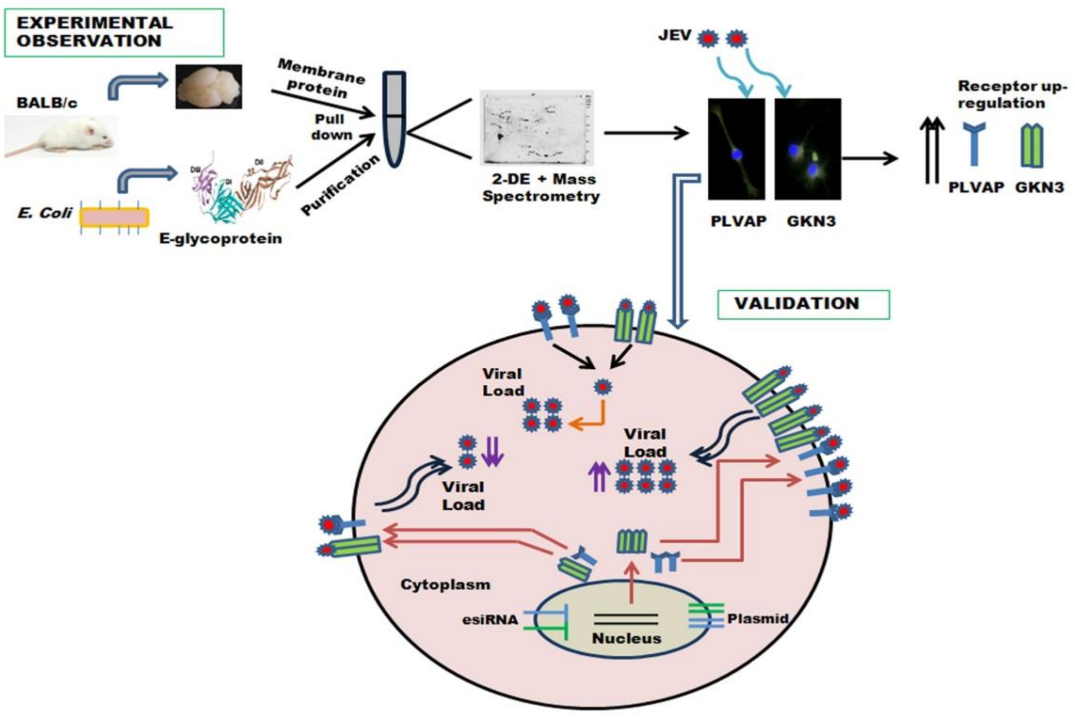

Supplement: Supplementary file 1 — Supplementary Information [file 41598_2018_30054_MOESM1_ESM.pdf]
